# Live *Brugia malayi* Microfilariae Inhibit Transendothelial Migration of Neutrophils and Monocytes

**DOI:** 10.1371/journal.pntd.0001914

**Published:** 2012-11-29

**Authors:** Jan-Hendrik Schroeder, Bigboy H. Simbi, Louise Ford, Sara R. Cole, Mark J. Taylor, Charlotte Lawson, Rachel A. Lawrence

**Affiliations:** 1 Royal Veterinary College, University of London, London, United Kingdom; 2 Liverpool School of Tropical Medicine, Liverpool, United Kingdom; Uniformed Services University of the Health Sciences, United States of America

## Abstract

Lymphatic filariasis is a major tropical disease caused by the parasite *Brugia malayi*. Microfilariae (Mf) circulate in the peripheral blood for 2–3 hours in synchronisation with maximal feeding of the mosquito vector. When absent from the peripheral blood, Mf sequester in the capillaries of the lungs. Mf are therefore in close contact with vascular endothelial cells (EC) and may induce EC immune function and/or wound repair mechanisms such as angiogenesis. In this study, Mf were co-cultured with human umbilical vein EC (HUVEC) or human lung microvascular EC (HLMVEC) and the transendothelial migration of leukocyte subsets was analysed. In addition, the protein and/or mRNA expression of chemokine, cytokine and angiogenic mediators in endothelial cells in the presence of live microfilariae were measured by a combination of cDNA arrays, protein arrays, ELISA and fluorescence antibody tests.

Surprisingly, our findings indicate that Mf presence partially blocked transendothelial migration of monocytes and neutrophils, but not lymphocytes. However, Mf exposure did not result in altered vascular EC expression of key mediators of the tethering stage of extravasation, such as ICAM-1, VCAM-1 and various chemokines. To further analyse the immunological function of vascular EC in the presence of Mf, we measured the mRNA and/or protein expression of a number of pro-inflammatory mediators. We found that expression levels of the mediators tested were predominantly unaltered upon *B. malayi* Mf exposure. In addition, a comparison of angiogenic mediators induced by intact Mf and *Wolbachia*-depleted Mf revealed that even intact Mf induce the expression of remarkably few angiogenic mediators in vascular EC. Our study suggests that live microfilariae are remarkably inert in their induction and/or activation of vascular cells in their immediate local environment. Overall, this work presents important insights into the immunological function of the vascular endothelium during an infection with *B. malayi*.

## Author Summary

*Brugia malayi* is a nematode which causes lymphatic filariasis in South and South-East Asia. Most infected people harbour many millions of the microfilarial stage of the parasite in their blood stream and yet they show few visible symptoms of disease. Vascular endothelial cells (EC) line the blood vessels and are therefore in direct contact with microfilariae. Since vascular EC are potent immune cells functioning in the production of both immune mediators and regulating the migration of immune cells from the blood into the tissue, we have established an *in vitro* model in which to test the effect of live Mf upon vascular EC function.

Strikingly, we observed that Mf exposure caused reduced transendothelial migration of neutrophils and monocytes, but not lymphocytes. However, microfilariae stimulated EC production of few pro-inflammatory mediators. Additionally, while filarial infection is known to stimulate mediators that increase blood vessel formation *in vivo*, live microfilariae promoted only a limited number of these regulators in cultured vascular EC. Our study suggests that the live microfilariae are remarkably inert in their induction and/or activation of vascular cells in their immediate local environment.

## Introduction

The filarial parasite *Brugia malayi* is a causative agent of human lymphatic filariasis in South and South-East Asia. *B. malayi* is transmitted by mosquitoes, which take up the blood-borne microfilarial stage (Mf) of the parasite. For the majority of the day, Mf sequester predominantly in the lungs of the host and they only appear in the peripheral blood circulation for a few hours, which coincides with maximal mosquito feeding [1], [2]. While sequestered in the lungs, *B. malayi* Mf are likely to interact with vascular endothelial cells (EC) and we have observed them binding to the surface of vascular EC (manuscript in preparation). Helminths are potent modulators of the immune response and filarial nematodes, in particular, have been shown to influence the secretion of inflammatory mediators from a number of different cell types [3], [4], [5]. Vascular EC themselves can modulate the immune response by producing pro-inflammatory cytokines and chemokines, in addition to several angiogenic mediators. Vascular EC also play a critical role in extravasation of leukocytes to the site of inflammation [6], [7].

To our knowledge no studies have addressed induction of local immune or inflammatory responses by vascular EC to live microfilariae of lymphatic filarial parasites. However, Bennuru *et al.* (2009) have shown that lymphatic EC (LEC) proliferate in response to adult, but not microfilarial, antigen and live parasites can induce tube formation by LEC in a contact-dependent manner. *B. malayi* microfilarial antigen also induced a number of angiogenic mediators in LEC. These data, together with an increased expression of angiogenesis and lymphangiogenesis mediators found in sera of humans infected with *Wuchereria bancrofti*, suggest that lymphatic filarial parasites may directly influence inflammation and angiogenesis [8], [9], [10]. Other helminths have been shown to induce pro-inflammatory mediators in EC, for example, *Schistosoma mansoni* schistosomulae stimulate production of the inflammatory cytokines, IL-6 and IL-7 [11], [12].

In this study, we investigated *B. malayi* Mf-induced immune responses in the local environment by modelling the interaction of Mf and vascular EC *in vitro*. Live *B. malayi* Mf directly inhibited extravasation of both neutrophils and monocytes, but not lymphocytes. However, Mf induced limited immune and angiogenic mediator expression. Several previous studies have shown that the filarial endosymbiotic bacteria, *Wolbachia* are partially responsible for induction of inflammatory and angiogenic mediators in filarial patients [8]. However, a comparison of angiogenic mediator mRNA expression induced by *Wolbachia*-depleted and live intact Mf, revealed that few angiogenic mediators were specifically induced by *Wolbachia* in vascular EC.

## Materials and Methods

### Ethics statement

Ethical approval was obtained from the East London Local Research Ethics Committee to collect human umbilical cords from mothers from the Royal London Hospital and blood from healthy donors. All study participants provided written informed consent. Parasites were obtained from infected animals in accordance with our Home Office project licence, which was approved under the Home Office (1986) Scientific Procedures Act.

### Human endothelial cell culturing

Human umbilical vein endothelial cells (HUVEC) were isolated from human umbilical cords using a modified previously published method [13]. In all experiments, HUVEC were used at passage 5. Cell morphology was confirmed by phase contrast microscopy. HUVEC were cultured in HUVEC medium (M199 supplemented with 150 U/ml penicillin, 150 U/ml streptomycin, 2 mM L-glutamine, 20% heat-inactivated FBS, 1 U/ml heparin and 0.03 mg/ml endothelial cell growth supplement from bovine neural tissue). Cryopreserved human lung microvascular endothelial cells (HLMVEC) were purchased from Clonetics (UK) and were cultured according to the supplier's recommendations. HLMVEC were used for experiments at passage 7–9.

### *B. malayi* microfilariae isolation

Infected gerbils (*Meriones unguiculatus*) were obtained from TRS Laboratories, Athens, Georgia, USA. Infection of gerbils was performed by i.p. injection of 400 *B. malayi* L3. *B. malayi* Mf were obtained by peritoneal lavage with RPMI-1640, 100–400 days post infection. Mf were isolated by centrifugation of recovered lavage fluid over lymphocyte separation medium (MP Biomedicals, USA).

To harvest *Wolbachia*-depleted Mf, gerbils were treated with tetracycline in their drinking water (2.5 mg/ml) for a period of 6 weeks [14]. Following treatment, Mf were isolated, genomic DNA extracted and the ratio of *Brugia* glutathione *S*-transferase (*gst*) to *Wolbachia* surface protein (*wsp*) copy numbers was measured by qPCR as previously described [15]. Using this measurement, the two batches of Mf isolated for use in *Wolbachia*-depletion experiments were shown to be 98.46% and 99.84% *Wolbachia*-free.

### Culturing of *B. malayi* Mf with HUVEC

1×10^6^ confluent HUVEC at passage 4 were cultured in HUVEC medium. After 60 hours of incubation the medium in each flask was replaced with co-culture medium (50% HUVEC medium (as above) plus 50% RPMI-1640 supplemented with 150 U/ml penicillin, 150 U/ml streptomycin, 2 mM L-glutamine, 20 mM HEPES, 20% heat-inactivated FBS and 20% of glucose solution) containing 125,000 *B. malayi* Mf. Co-culture medium without *B. malayi* Mf was added to HUVEC in control flasks. After 24 hours of co-culture, EC or the EC supernatant were collected for further investigation. In some experiments, EC were stimulated with 10 ng/ml IFN-γ (ImmunoContact, USA) for 24 or 48 hours prior to co-culture with Mf. When HLMVEC were co-cultured with Mf, 50% EGM-2 MV BulletKit medium (Clonetics) was used in place of HUVEC medium.

### Peripheral blood mononuclear cells and granulocyte isolation

With approval from East London Local Research Ethics Committee whole human blood was collected in 20 U/ml heparin. Peripheral blood mononuclear cells (PBMC) were isolated using lymphocyte separation medium. The intermediate layer of PBMC was collected, washed twice and re-suspended in complete RPMI-1640 and 10% FBS.

To isolate granulocytes, the pellet remaining from the lymphocyte separation medium was re-suspended in a 50∶50 mix of RPMI-1640/10% FBS and 0.9% NaCl. The final solution was supplemented with 3% dextran. After one hour the upper layer was removed and centrifuged at 129×g for 10 minutes at 4°C. The pellet was re-suspended in ice cold 0.2% NaCl for 30 seconds. An equal volume of ice cold 1.6% NaCl was added and the mixture was centrifuged at 129×g for 6 minutes at 4°C. This process was repeated until the cell pellet was free of red blood cells. After the final wash, granulocytes were re-suspended in RPMI-1640 supplemented with 10% FBS and kept on ice until use.

### Transmigration and chemotaxis assays

1×10^5^ HUVEC were added to human fibronectin-coated cell culture inserts in the wells of a 24-well plate (Greiner Bio-One). 6 h later HUVEC were stimulated with human TNF-α at a concentration of 20 ng/ml. After another 18 h, 50% medium was removed from each transwell and replaced with complete RPMI-1640 20% FCS 20% glucose solution supplemented with 12,500 Mf. In control conditions, no Mf were added. After another 24 h, 50% of medium was removed from each transwell and replaced with RPMI-1640 supplemented 10% FBS and either 1×10^6^ PBMC or 1×10^6^ granulocytes. RPMI-1640 plus 10% FBS was added into the lower wells. After 4 hours transmigrated cells were harvested from the lower wells and analysed by flow cytometry and/or cytospin.

For cytospin analyses, cells were centrifuged in a cytospin at 800×g for 5 minutes. The slides were fixed with 50% acetone: 50% methanol for 2 minutes and stained with May-Gruenwald stain for 10 minutes. Cell morphology was examined by phase contrast microscopy. Granulocytes transmigrating through the endothelial monolayer were 100% neutrophils.

For chemotaxis experiments, unstimulated HUVEC were co-cultured with Mf in the lower well and after 24 h either 1×10^6^ PBMC or granulocytes were added in RPMI-1640 supplemented with 10% FBS into the upper well. After 4 h, cells that had migrated into the lower well were analysed by flow cytometry.

### Antibodies and flow cytometry

Mouse anti-human CD8 antibodies were prepared by growing the OKT8 hybridoma in RPMI-1640/10% FBS *in vitro*. Supernatant was harvested after 7 days and centrifuged at 2,057×g for 10 minutes. Antibodies were purified over protein G sepharose.

Mouse anti-human CCR5 (BD), mouse anti-human CD14 (26ic) (in-house), mouse anti-human CD8 (OKT8) (in-house), PE-conjugated mouse anti-CD56 (eBioscience), FITC-conjugated mouse anti-human CD3 (eBioscience), PE-Cy5-conjugated mouse anti-human CD16 (BD Pharmingen) and the isotype control antibodies FITC–conjugated mouse IgG1 (BD Pharmingen), PE-Cy5–conjugated mouse IgG1 (eBioscience) and PE–conjugated IgG2a (BD Pharmingen) were used to stain cells. Goat anti-mouse IgG FITC conjugated antibodies (Sigma) were used as a secondary antibody with unconjugated primary antibodies. Negative control samples for unconjugated primary antibodies were solely stained with this secondary antibody. Data was acquired using a FACS Canto II (BD Oxfordshire UK) and analysed with FlowJo software (Tree Star Incorporation).

### Preparation of EC protein lysate

HUVEC were washed twice with ice-cold PBS. Cells were lysed using RIPA Buffer (20 mM MOPS, 150 mM NaCl, 1 mM EDTA, 1% Igepal, 1% Sodium deoxycholate and 0.1% SDS supplemented with a 1∶1000 concentration of protease inhibitor mix (Sigma)). Genomic DNA was broken up by mechanical syringe action. The lysates were centrifuged at 10,400×g for 10 minutes at 4°C. The supernatant was kept at −80°C until use. The protein concentration was measured using a BCA protein assay (Pierce).

### Protein array

The cytokine protein levels in supernatants were analysed using protein arrays (RayBioTech) according to the manufacturer's instructions. The dot intensity on the membranes when exposed to X-ray film was measured using QuantityOne Software (BioRad Laboratories). Subsequently, the levels of cytokines were analysed using the RayBio Analysis Tool for the human cytokine antibody array I (RayBioTech).

### SDS electrophoresis and Western blotting

Protein expression was detected in cell lysates by SDS-PAGE followed by Western blotting. Blots were incubated with mouse anti-human heme oxygenase-1 (HO-1) (BD transduction Laboratories) or mouse anti-human β-actin antibodies. HRP-conjugated rabbit anti-mouse (Dako) antibody was used for detection and developed with SuperSignal West Pico Chemiluminescent Substrate (Pierce).

### Cytokine ELISA

ELISA was used to measure human IL-1β, TNF-α, IL-6, IL-8, CCL2, TGF-β1 (R&D Systems) and IL-13 (Pelikine Compact) in EC supernatant or lysate according to the manufacturer's instructions.

### Oligo microarrays

Total EC RNA was harvested using QIAShredder and RNeasy kit as advised by the manufacturer (Qiagen, Brighton UK). RNA quantity and quality were evaluated using the NanoDrop ND-1000 spectrophotometer. Total RNA integrity was verified using agarose gel electrophoresis and ensuring that 18 and 28S ribosomal RNA bands were intact.

Oligo microarrays for chemokines and chemokine receptors (Supp. Table 1–2), and angiogenesis mediators (Supp. Table 5) were purchased from SuperArray Bioscience (UK) and were also used according to the manufacturer's instructions. The dot intensity of the oligo microarrays when exposed to X-ray film was measured using GE Array Analysis Suite (SuperArray Bioscience). Sample values were considered to be different, if both values from two duplicate experiments were either lower or higher in gene expression units than the comparative samples, and if the means of the duplicates differed by at least a factor of 4 in gene expression units. The reference value for β-actin in these experiments was 1 (chemokine & chemokine receptors) or 11 (angiogenesis mediators) gene expression units.

### Real-time quantitative RT-PCR

cDNA was synthesised from 1 µg total RNA using the QuantiTect Reverse Transcription kit (Qiagen) following the manufacturer's instructions. Primer sequences used and Entrez accession numbers for each gene are outlined in Supp. Table 4. Primers were designed using the Primer-3 Web-Software (Whitehead Institute for Biomedical Research, MA, USA) and purchased from MWG-Biotech (Ebersberg, Germany). Real-time qRT-PCR was performed as previously published [16] and quantification analysis was carried out using the MJ Research Opticon 3.1 software from standard curves with correlation coefficient (r^2^) greater than 0.98. Gene expression data was normalised to total RNA and presented as copy numbers. Specificity and purity of amplificons were verified from melting curves and agarose gel electrophoresis.

### Statistics

The Students t-test for paired data was used in all statistical analyses and performed with Prism 4 software (GraphPad Software, Inc). P values<0.05 were taken to be statistically significant. All data are presented as mean ± standard deviation.

## Results

### Live Mf inhibit the transendothelial migration of monocytes and neutrophils

**Figure 1:**
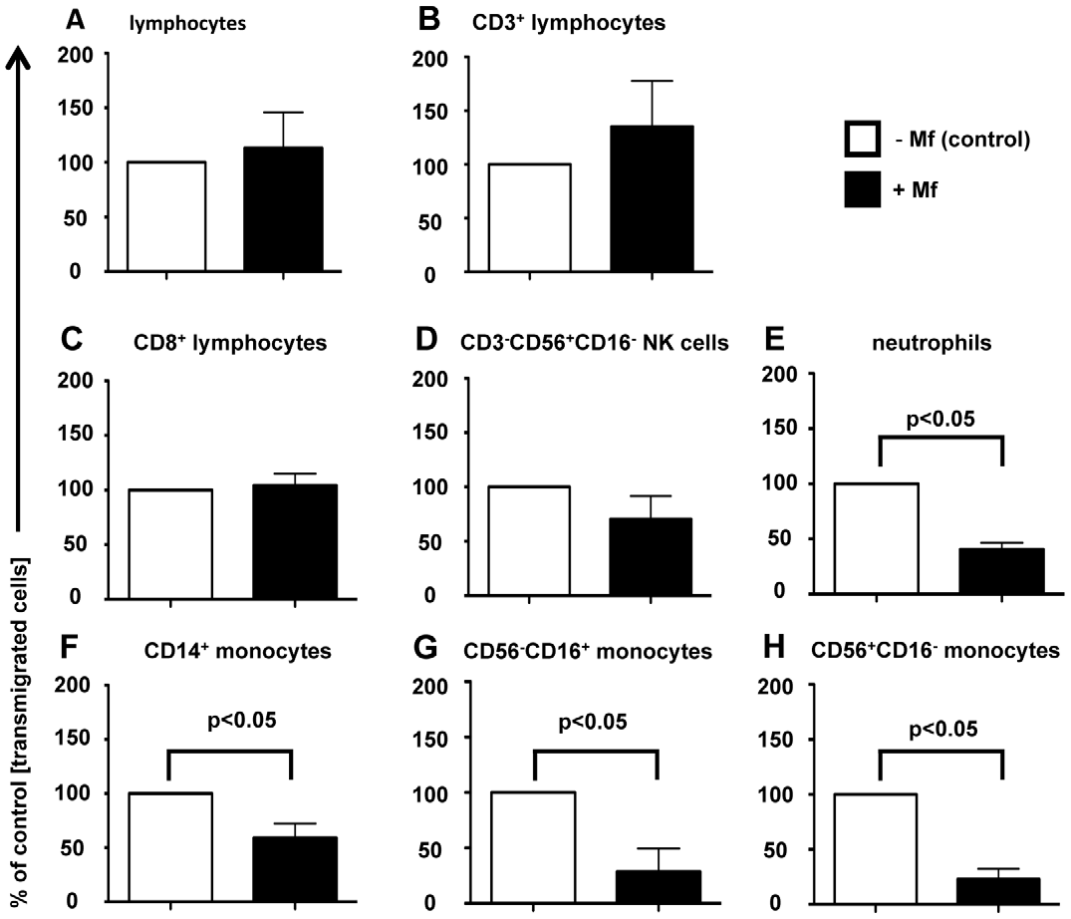
Transendothelial migration of monocytes and neutrophils, but not lymphocytes, is inhibited in the presence of live Mf. TNF-α-prestimulated HUVEC were co-cultured with or without live Mf in inserts of a transmigration assay plate. After 24 hours, 1×10^6^ PBMC or granulocytes were added into the inserts. A further 4 hours later, transmigrated PBMC or granulocytes were harvested from the lower wells and the number of transmigrated (a) total lymphocytes, (b) CD3^+^ lymphocytes, (c) CD8^+^ lymphocytes, (d) CD3^−^CD56^+^CD16^−^ NK cells, (e) neutrophils, (f) CD14^+^ monocytes, (g) CD3^−^CD56^+^CD16^−^ monocytes and (h) CD3^−^CD56^+^CD16^+^ monocytes were analysed by flow cytometry. Total lymphocytes and neutrophils were defined by FSC:SSC gates of transmigrated cells. The percentage of cells that transmigrated in the presence of Mf was compared to extravasation in Mf absence. Data are shown as the mean and standard deviation per group of eight (a), three (b–d, g–h) or six (e–f) independent experiments. p values were determined using the Student's t test.

Presence of *B. malayi* microfilariae in the blood vessels may alter transmigration of leukocytes across the vascular endothelium. In order to investigate this, TNF-α - stimulated HUVEC were co-cultured with or without live Mf in transwells of a transmigration assay plate and the ability of PBMC or neutrophils to transmigrate through the confluent HUVEC monolayer was analysed. The presence of live Mf did not affect the total number of extravasated lymphocytes, CD3^+^ (T cells), CD8^+^ cells or CD3^−^CD56^+^CD16^−^ (NK cell subset) (Figure 1a–d). However, the transendothelial migration of neutrophils and CD14^+^, CD3^−^CD56^−^CD16^+^ and CD3^−^CD56^+^CD16^−^ monocytes was significantly inhibited in the presence of live Mf (p<0.05) (Figure 1e–h).

**Figure 2:**
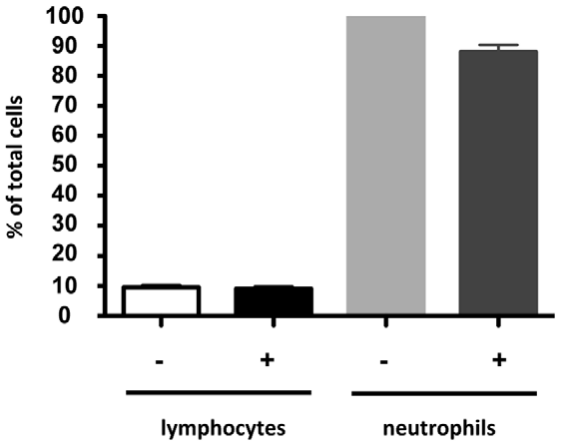
Live Mf presence does not alter chemotaxis of lymphocytes and neutrophils. HUVEC were co-cultured with or without live Mf in the lower well of a transmigration assay plate. After 24 hours, PBMC or granulocytes were added in the inserts. After a further 4 hours leukocytes attracted into the lower wells were analysed using flow cytometry. Data is shown as the mean and standard deviation of an experimental triplicate.

To investigate whether Mf altered the chemotactic ability of leukocytes and/or the ability of vascular EC to chemoattract, the chemotaxis of lymphocytes and neutrophils to HUVEC in the presence of Mf was analysed. Interestingly, neutrophils were more strongly attracted to HUVEC than lymphocytes, however Mf presence did not significantly alter the chemotactic ability of either leukocyte subset (Figure 2).

### Live *B. malayi* Mf do not stimulate protein secretion of key endothelial cell-derived cytokines or chemokines

**Figure 3:**
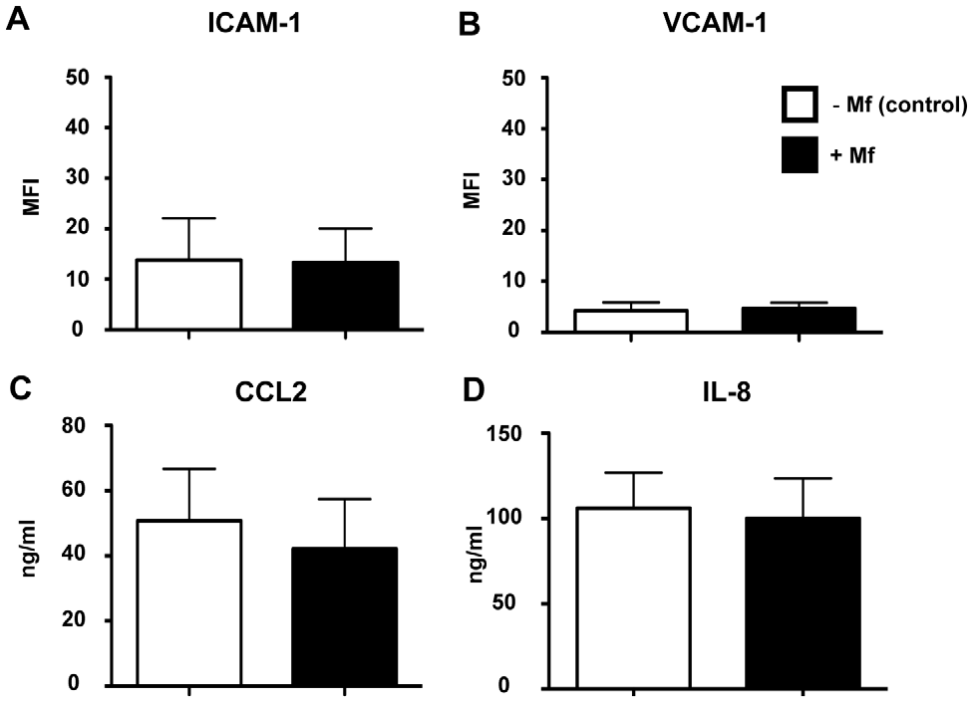
Live Mf presence does not alter HUVEC expression of key mediators of the tethering stage of monocyte and neutrophil extravasation. HUVEC were cultured with or without Mf for 24 hours prior to flow cytometry analysis and harvesting of supernatant. HUVEC surface expression of (a) ICAM-1 and (b) VCAM-1 was analysed by flow cytometry. Using ELISA, the supernatant was analysed for the protein secretion of (c) CCL2 and (d) IL-8. Data are shown as the mean and standard deviation per group of three independent experiments.

Altered expression of adhesion molecules and/or chemokines by vascular EC may inhibit the extravasation of leukocytes. To further investigate the mechanism of reduced monocyte and neutrophil extravasation in Mf presence, the key adhesion molecules, ICAM-1 and VCAM-1, expressed by HUVEC were measured. The presence of *B. malayi* Mf did not alter the surface expression of these adhesion molecules (Figure 3a–b). The chemokines, CCL2 (MCP-1) and IL-8 are potent inducers of monocyte and neutrophil extravasation, respectively. In endothelial cell biology the amount of chemokine secreted corresponds to the level of chemokine presented at the vascular surface. Therefore, CCL2 and IL-8 were measured by ELISA, in the supernatants of HUVEC cultured with or without Mf. However, the presence of *B. malayi* Mf did not have a significant effect on the up- or down-regulation of either CCL2 or IL-8 (Figure 3c–d).

### Live *B. malayi* Mf do not significantly alter the mRNA or protein expression of key EC chemokines and cytokines

**Figure 4:**
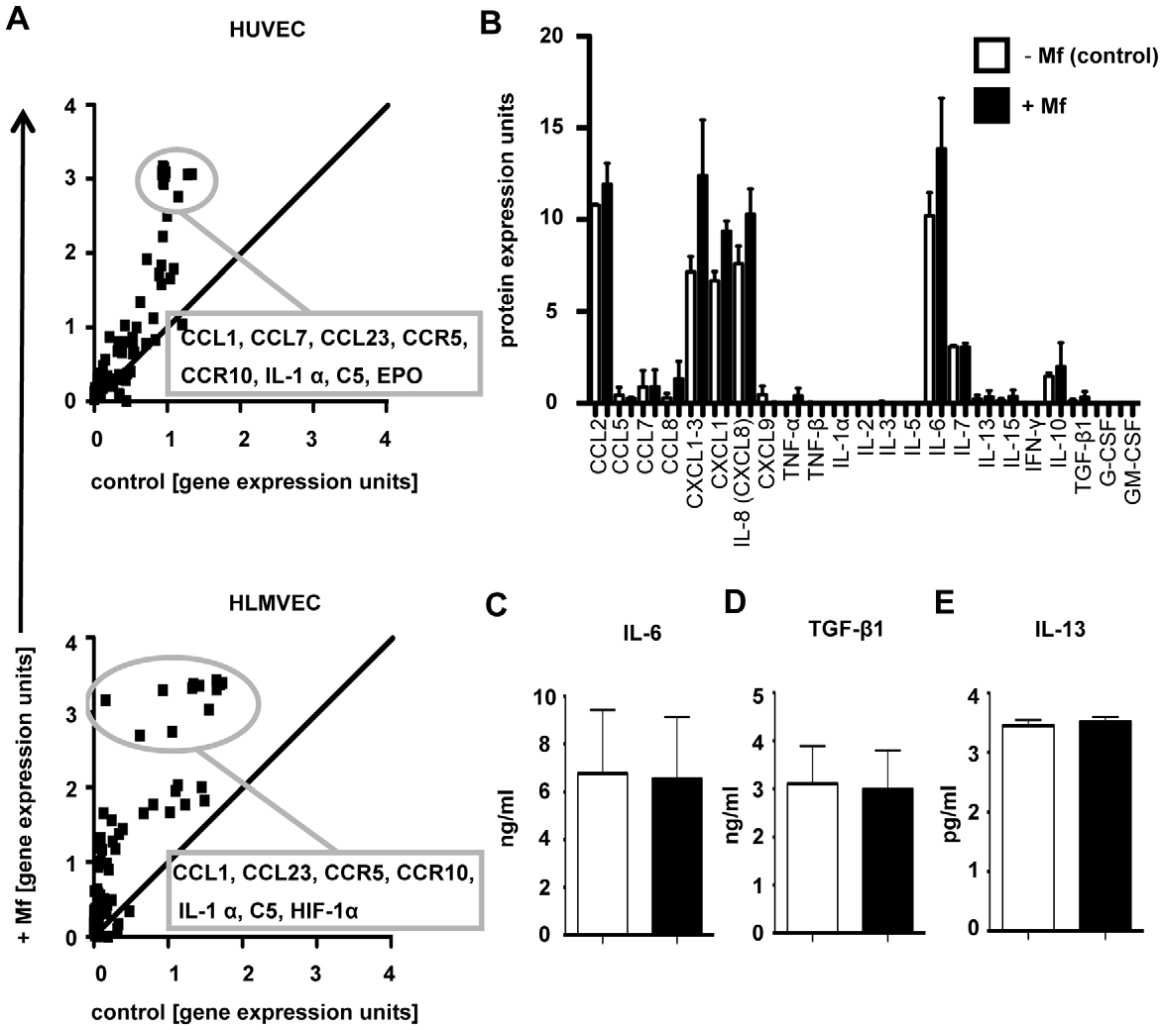
Live *B. malayi* Mf presence does not alter gene expression of vascular EC cytokine, chemokine and chemokine receptors. HUVEC or HLMVEC were cultured with live *B. malayi* Mf for 24 hours prior to isolation of total EC RNA, harvest of supernatant or cell lysis. An oligo microarray was used to analyse mRNA expression of various cytokines, chemokines and chemokine receptors. Genes with the greatest differences in expression upon Mf exposure are circled in (a). Oligo microarray data are shown as the mean of two independent experiments. HUVEC supernatant was analysed for protein expression of various cytokines using a protein array (b) or the cytokines IL-6 (c) and TGF-β1 (d) were measured by ELISA. IL-13 was measured in HUVEC lysate by ELISA (e). Protein assays show the mean and standard deviation per group of two (protein arrays) or three (ELISA) independent experiments. The label CXCL1-3 in the protein array defines the measurement of a common epitope for CXCL1, CXCL2 and CXCL3.

To further investigate whether *B. malayi* Mf alter the EC expression of immune mediators in their immediate environment, a comprehensive analysis of cytokine and chemokine mRNA expression was performed by oligo microarray (Figure 4a, Supp. Table 1 and 2). Since Mf are situated in the lung capillaries for long periods of time, in addition to HUVEC, we also used HLMVEC, as the latter may more closely resemble EC in the locality of Mf *in vivo*. Within the stringency criteria of our experiments, neither cytokine nor chemokine mRNA expression in vascular EC was found to be altered in live Mf presence (Figure 4a). However, the mediators with the highest fold increases in mRNA expression in the presence of Mf were almost identical between the two different vascular EC, HUVEC and HLMVEC. These mediators were CCL1, CCL23, IL-1α, C5, and the chemokine receptors CCR5 and CCR10 (Figure 4a).

To investigate whether live Mf stimulate and/or down-regulate the immune function of vascular EC, secretion of pro- and anti-inflammatory cytokines and chemokines was measured in the supernatants of HUVEC following exposure to Mf (Figure 4b–d). Initially, an exhaustive exploration of cytokines and chemokines produced by HUVEC in the presence of *B. malayi* Mf, was conducted by protein expression array in culture supernatants (Figure 4b, Supp. Table 3). Mf presence appeared not to significantly alter the secretion of any of the immune mediators tested. Indeed the array confirmed our previous results that CCL2 and IL-8 are not altered in Mf presence (Figure 3c–d and 4b). Although high levels of GRO family members (CXCL1, CXCL2, CXCL3) were detected, expression of these chemokines was not significantly enhanced by Mf.

Key inflammatory cytokines known to be produced by EC were also measured by ELISA (Figure 4c–d, and data not shown). In confirmation of the protein array data, the secretion of IL-6, TGF-β1, TNF-α and IL-1β by HUVEC was not altered in Mf presence. Indeed, IL-1β and TNF-α were not detected in the HUVEC supernatant in the presence or absence of Mf (data not shown). IL-13 was not found in HUVEC supernatants but was detected in HUVEC lysates by ELISA (Figure 4e), however, live Mf did not alter the protein expression of this cytokine.

### qRT-PCR analysis of EC mediator expression upon exposure to Mf

**Figure 5:**
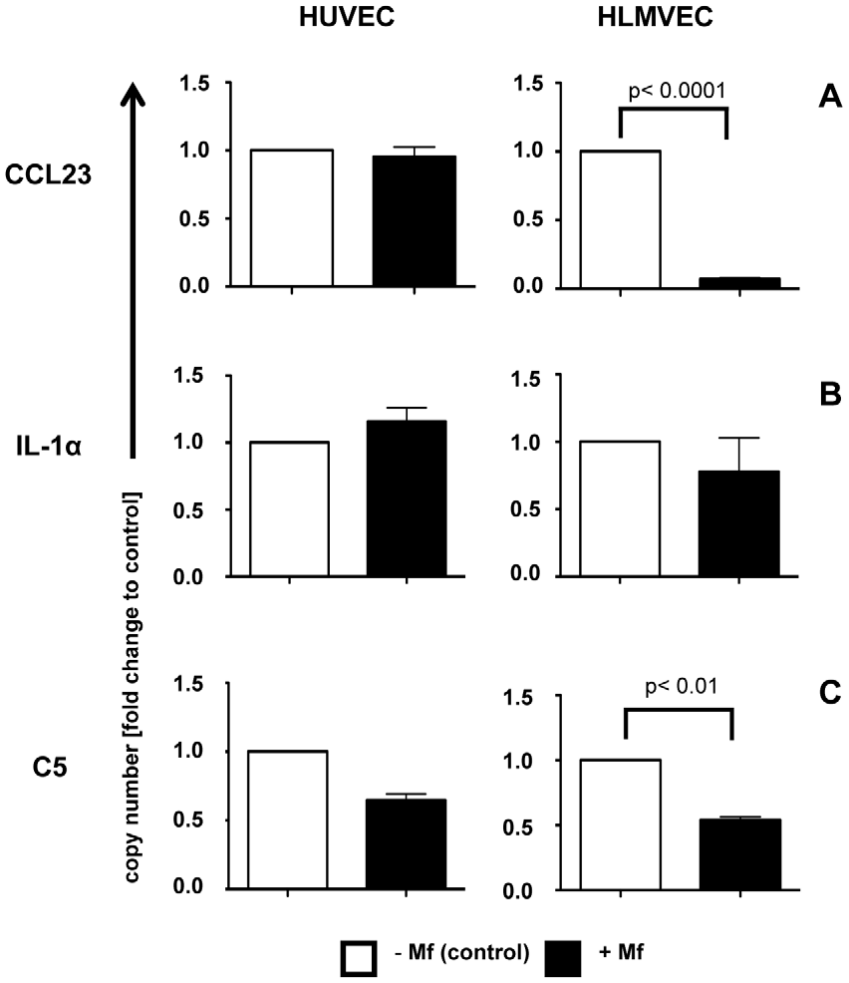
Live *B. malayi* Mf presence does not greatly alter gene expression of vascular EC pro-inflammatory mediators. HUVEC or HLMVEC were cultured with live *B. malayi* Mf for 24 hours prior to isolation of total EC RNA. Real time quantitative RT-PCR was used to analyse mRNA expression of (a) CCL23, (b) IL-1α and (c) C5. Data are shown as the mean and standard deviation per group of three independent experiments and p values were determined using the Student's t test.

While the oligo microarray analysis did not show any mediators significantly up- or down-regulated in HUVEC or HLMVEC in the presence of Mf; we sought to more definitively determine whether the mediators with highest mRNA expression levels were altered by Mf. Therefore, we used qRT-PCR to analyse the mRNA levels of selected genes. In accord with the oligo microarray data and the applied analysis criteria, qRT-PCR confirmed that HUVEC mRNA expression of CCL1 (not detected), CCL23 and IL-1α were not altered by Mf presence (Figure 5a–b). In addition, Mf presence caused no alteration in CCL1 (not detected) and IL-1α mRNA expression in HLMVEC (Figure 5b). However, CCL23 mRNA was significantly (p<0.0001) downregulated upon Mf exposure. Interestingly, Mf presence also caused a down-regulation of the mRNA levels of pro-inflammatory C5 in both HUVEC and HLMVEC as analysed by qRT-PCR (Figure 5c).

**Figure 6:**
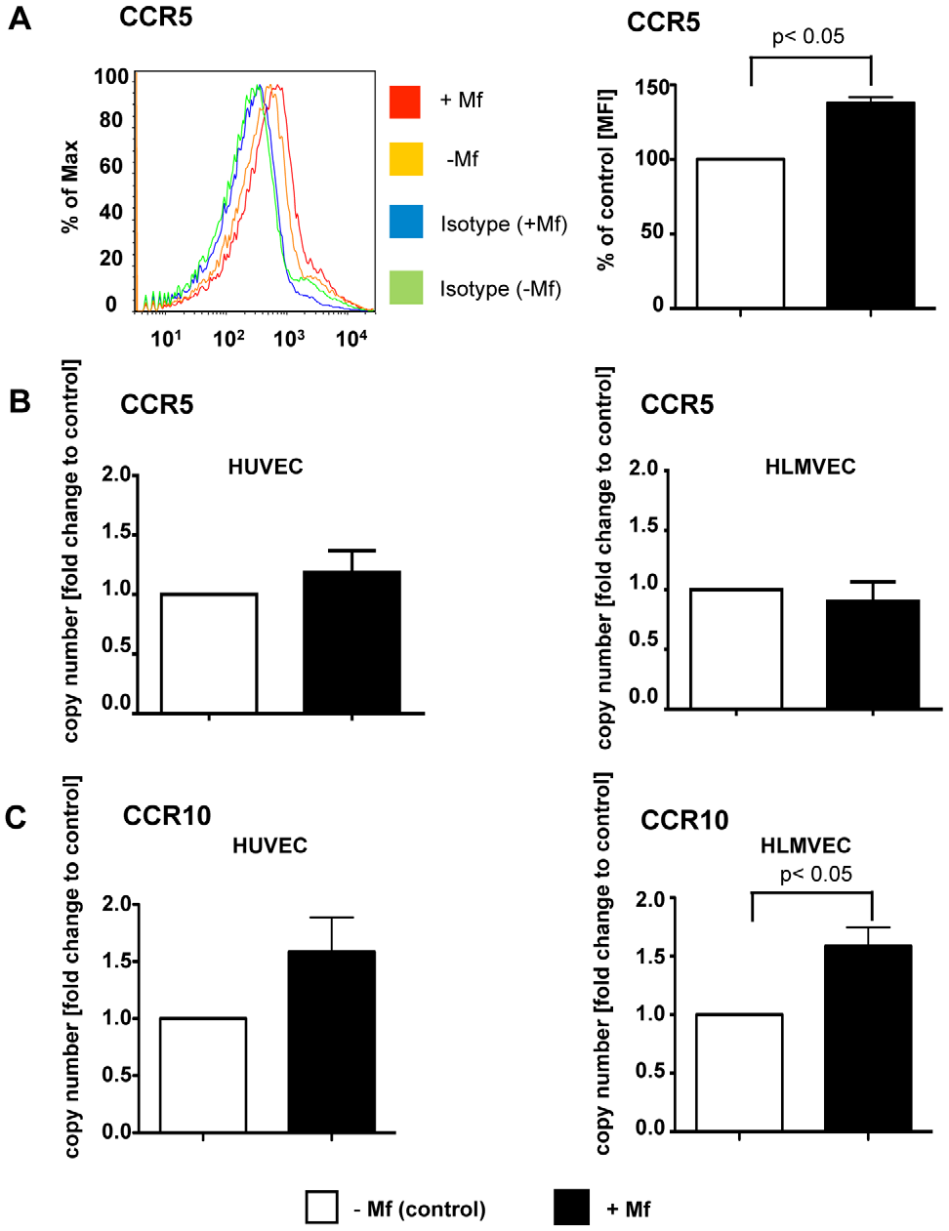
CCR5 and CCR10 are upregulated in vascular EC by live Mf presence. HUVEC or HLMVEC were cultured with live *B. malayi* Mf for 24 hours prior to isolation of total EC RNA and flow cytometric analysis. CCR5 surface expression was measured by flow cytometry of HUVEC incubated with or without Mf and labeled with isotype control or anti-human CCR5. The FACS plot is presented and a bar graph presenting mean fluorescence intensity (MFI) of CCR5 in HUVEC ± Mf (a). Real time quantitative RT-PCR was used to analyse mRNA expression of CCR5 (b) and CCR10 (c) in HUVEC and HLMVEC. Data are shown as the mean and standard deviation of three independent experiments and analysed by Students t-test.

Expression of the chemokine receptors, CCR5 and CCR10, were also further analysed in both HUVEC and HLMVEC exposed to live Mf (Figure 6a–c). Initially, HUVEC co-cultured with or without *B. malayi* Mf were analysed by flow cytometry for surface expression of CCR5 (Figure 6a). CCR5 was found to be significantly upregulated on the surface of HUVEC exposed to live Mf (p<0.05). However, mRNA expression analysis of HUVEC and HLMVEC CCR5 by qRT-PCR did not show alteration in the presence of live Mf. Furthermore, qRT-PCR for CCR10 revealed that live Mf increased mRNA in both HUVEC and HLMVEC, however this was only significant in HLMVEC (p<0.05) (Figure 6c). The oligo microarray analysis of other chemokine receptors revealed no other differences in mRNA expression in either HUVEC or HLMVEC in the presence of Mf (Figure 4a). In some instances therefore, discrepancies existed between the recognition of mRNA by the primers used in qRT-PCR and the sensitivity of the probes used on the oligo-microarray.

### Live *B. malayi* Mf induce only limited mRNA expression of pro-angiogenic mediators in vascular EC

Live Mf could be responsible for increased levels of pro-angiogenic mediators found in the sera of filarial patients [8], [9]. Furthermore, previous studies have suggested that the filarial endosymbiotic bacteria, *Wolbachia*, may induce angiogenesis [8].

**Figure 7:**
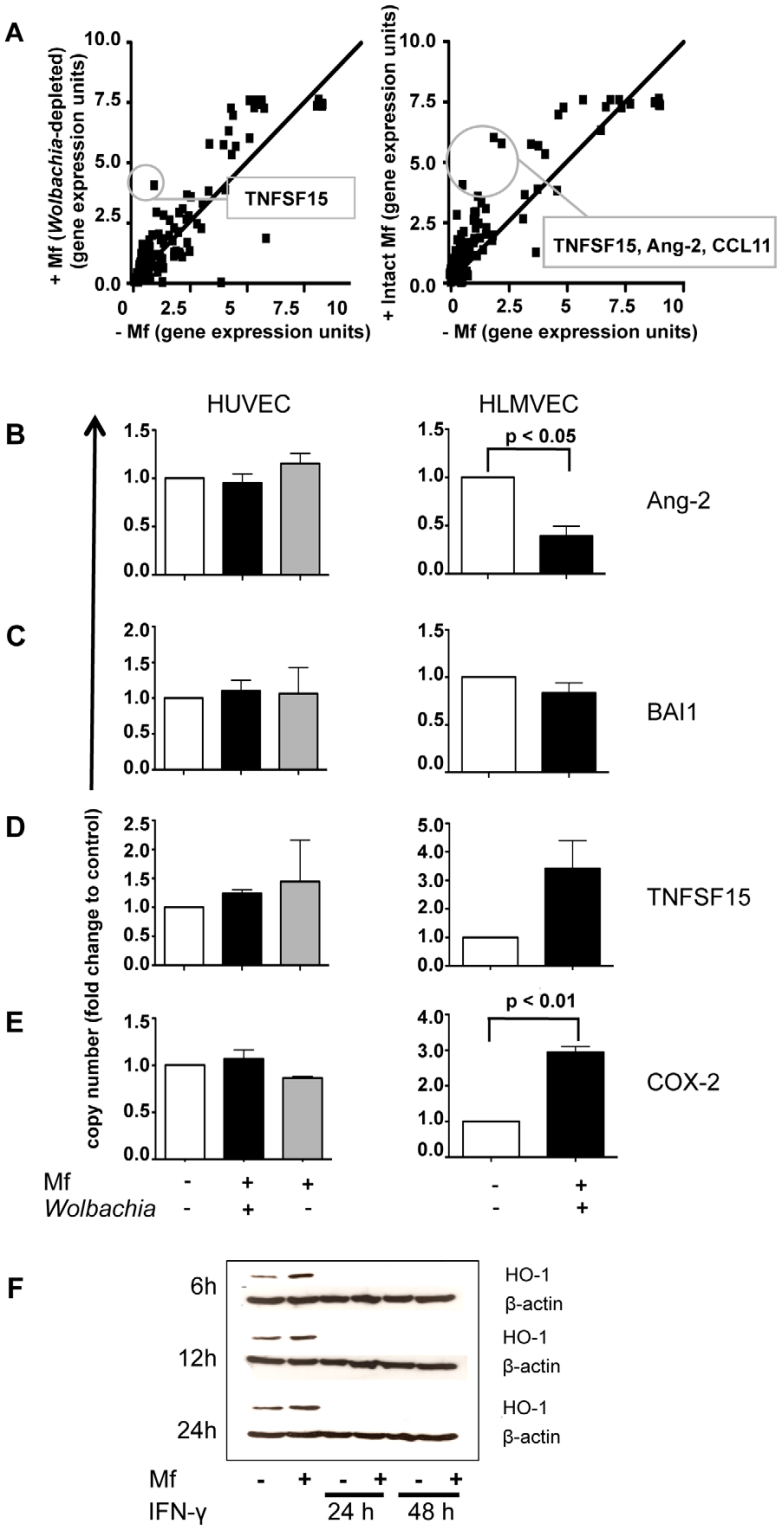
Limited alterations in the gene expression of angiogenic mediators in vascular EC in the presence of live *B. malayi* Mf. HUVEC or HLMVEC were cultured with or without live untreated *B. malayi* Mf or *Wolbachia*-depleted Mf for 24 hours prior to isolation of total EC RNA. An oligo microarray was used to analyse mRNA expression of various angiogenesis mediators (a). mRNA expression was compared between HUVEC in the absence of Mf vs. presence of intact Mf and HUVEC in the absence of Mf vs. presence of *Wolbachia*-depleted Mf. HUVEC and HLMVEC mRNA expression of (b) Ang-2, (c) BAI-1, (d) TNFSF15 and (e) COX-2 was also analysed by real time qRT-PCR. Data are shown as the mean of two independent experiments (oligo microarray) or mean and standard deviation of three independent experiments (qRT-PCR). Students t-test was used to analyse statistical significance. (f) HO-1 protein expression in HLMVEC is enhanced by live Mf presence. HLMVEC were left unstimulated or were stimulated with IFN-γ for 24 or 48 hours, prior to co-culture with live Mf for a further 6, 12 or 24 hours. HLMVEC cells were lysed and the cell lysate was analysed for protein levels of HO-1 by western blotting.

In order to investigate whether live Mf induce angiogenic mediators in EC, the mRNA expression of these mediators in HUVEC and HLMVEC following co-culture with *B. malayi* Mf was analysed (Figure 7, Supp. Table 5). In addition, to determine whether *Wolbachia* endosymbionts are responsible for any angiogenic mediator induction, oligo microarray of mRNA from EC cultured with either intact Mf or Mf-depleted of *Wolbachia* were compared in two separate experiments. These studies showed that angiogenic factors were not altered in HUVEC in the presence of either intact Mf or *Wolbachia*-depleted Mf (Figure 7a). A qRT-PCR analysis of several mediators with the highest fold change expression in Mf presence (angiopoietin-2 (Ang-2), brain-specific angiogenesis inhibitor-1 (BAI-1), tumor necrosis factor superfamily member 15 (TNFSF15), cyclooxygenase-2 (COX-2) and CCL11), confirmed these results (Figure 7b, Supplementary Figure 1). However, in HLMVEC, Ang-2 mRNA was downregulated and the angiostatic factor TNFS15 was upregulated by live Mf (Figure 7b,d). Further analysis of the pro-angiogenic mediator COX-2, showed that this mediator was upregulated in HLMVEC, but not HUVEC, by Mf presence (Figure 7b).

Live *B. malayi* Mf. enhanced, protein expression of the hypoxia-induced product, heme oxygenase-1 (HO-1), in HLMVEC after 6, 12 and 24 h of co-culture with Mf (Figure 7f). Following IFN-γ-stimulation, HO-1 was no longer detectable at any of these time points. Interestingly live Mf also induced relatively high levels of hypoxia-inducible factor (HIF-1α) mRNA in HLMVEC (Figure 4a). Presence of HIF-1α is an indicator of hypoxia which in turn is a potent promoter of angiogenesis.

## Discussion

In an area endemic for lymphatic filariasis, the majority of people have asymptomatic infection and harbour several million Mf in their blood stream. Vascular EC play an important role in mediating immune and angiogenic responses. Therefore, maintenance of this asymptomatic condition, as well as survival of *B. malayi* Mf in the blood stream, could depend upon Mf-driven modulation of EC activity. In this study we sought to investigate the vascular EC response upon exposure to live *B. malayi* Mf. We found that the transendothelial migration of monocytes and neutrophils, but not lymphocytes, is inhibited by live Mf presence; while either intact or *Wolbachia*-depleted Mf stimulate few cytokines, chemokines or angiogenic mediators.

Both macrophages and neutrophils are capable of killing *B. malayi* Mf *in vitro* [17], [18], [19]. Reduced extravasation of monocytes and neutrophils could therefore lead to retention of effector leukocytes in the vascular location of the parasite, resulting in increased clearance of Mf. Indeed, reduced eosinophil extravasation, in eotaxin-1^−/−^ mice infected with *B. malayi* Mf, lead to eosinophil retention in the blood stream and enhanced Mf clearance [20]. Both monocytes and neutrophils appear to kill Mf via production of reactive intermediates, however, in turn, Mf can partially neutralise the toxic effects of these intermediates by secreting anti-oxidant enzymes such as peroxidases and superoxide dismutase [17], [21], [22], [23].

There are a number of potential mechanisms, which could result in the inhibition of monocyte and neutrophil transendothelial migration in the presence of Mf. In general, leukocyte extravasation is a multi-step cascade involving rolling mediated by selectin-selectin ligand axes, tethering mediated by integrin-adhesion molecule axes strengthened by chemokine-triggered activation and finally, diapedesis [24]. Selectin-selectin ligand axes are unlikely to have a functional role in the static transendothelial migration experiments performed in this study. Furthermore, HUVEC surface expression of the adhesion molecules ICAM-1 and VCAM-1, which have crucial roles at the tethering step, was not modulated upon Mf exposure. Another potential mechanism investigated was the possibility that alteration(s) in chemokine expression in the presence of Mf selectively interfered with leukocyte tethering.

IL-8 and CCL2 are considered to be the most important chemokines for the transendothelial migration of neutrophils and monocytes respectively [25]. However, Mf presence had no effect on IL-8 or CCL2 production by EC. In accord with this, a comprehensive examination (by oligo microarray, qRT-PCR and protein array) of chemokines in EC exposed to Mf, did not reveal any major differences in these or other chemokines that may have a role in extravasation of monocytes and neutrophils. No alteration in the transendothelial migration of whole T cells, CD8^+^ cells or NK cells was observed in Mf presence.

Interestingly, in agreement with our study, experiments in mice implanted with adult *B. malayi* or Mf *in vivo* showed that in the presence of Mf alone, infiltration of leucocytes into the peritoneal cavity is reduced in comparison to adult nematode implanted mice [26]. In addition, the proportion of macrophages within these leukocyte populations was significantly lower in Mf implanted rather than adult implanted mice [26]. Previous work has also shown that a serine protease derived from *B. malayi* Mf abolishes C5a-mediated chemotaxis of granulocytes [27]. Furthermore both adult *B. malayi* and Mf extracts inhibit hyper-permeability induced by TNF-α or IL-1α, of lymphatic EC monolayers to dextran. Although neither extract showed any effect on the permeability of confluent EC *per se* [10]. *Dirofilaria immitis* adult extracts, however, did reduce the transendothelial permeability of a human EC line. Enhanced expression of tight junction and/or adherence molecules by both *Brugia* and *Dirofilaria* extracts has been shown to be the likely mechanism of this reduced permeability [10], [28], [29]. Indeed, *Wolbachia* surface protein (WSP), but not whole *D. immitis* extract, induced the expression of ICAM-1 and VCAM-1 on a human EC line [28], and, WSP or *D. immitis* extracts up-regulated CD31 on this EC line [28], [29]. Strikingly, lymphatic EC exposed to *Brugia* adult or Mf extract also had higher mRNA levels of CD31, in addition to, VE-cadherin and Junctional Adhesion Molecule-C (JAM-C) [10]. If live Mf presence also causes elevated expression of these intercellular adhesion molecules, this may provide an explanation for the retention of monocytes and neutrophils while the transmigration of smaller lymphocytes is not affected.

In addition, we investigated whether live *B. malayi* Mf initiate immune responses in their local environment. Perhaps, not surprisingly, live *B. malayi* Mf (as opposed to extracts [10], [21]) appear to be relatively inert in their local vascular environment and do not induce significant levels of pro-inflammatory immune mediators from EC, such as IL-6, TNF-α or IL-1β. Interestingly, Mf also did not induce increased levels of IL-13, which promotes alternatively-activated macrophages (AAMø), or the down-regulatory cytokines IL-10 or TGF-β1. However, Mf presence did down-regulate mRNA expression of the inflammatory complement component, C5, in HUVEC and HLMVEC. In light of the recent report, that *B. malayi* Mf secrete a C5a-cleaving serine protease, this suggests that C5 products may be potentially damaging to filarial nematodes [27]. Similarly, mRNA expression of CCL23, a chemoattractant for monocytes, neutrophils and T cells, was downregulated in HLMVEC. Both of these latter observations indicate that Mf may modulate inflammatory responses. This is in accord with the fact that most filarial patients are asymptomatic and have down-regulated cellular immune responses to filarial antigens, however, when given therapeutic treatments, patients subsequently regain responses to filarial antigen [30]. This also suggests that while the inflammatory potential of *B. malayi* is dependent on the presence of *Wolbachia* [31], [32], *Wolbachia* and/or their products are not released or secreted from living Mf to induce inflammatory mediators in their local environment. However upon death of worms, *Wolbachia* and their inflammatory products such as lipoprotein, which has been shown to stimulate both innate and adaptive immunity are released [31], [33], [34].

Interestingly, we observed that Mf exposure induced upregulation of the hypoxia-responsive mediator HO-1 in HLMVEC, indicating that Mf may induce hypoxia, which is an angiogenesis-promoting condition. Furthermore in HLMVEC Mf upregulated mRNA for hypoxia-inducible factor (HIF-1α) which is known to induce HO-1. In addition to hypoxia and HIF-1, HO-1 can be induced by other components such as heme, IL-6, IL-1 or LPS in a number of model systems [35], [36], [37], [38], [39], [40], and is often used as a marker of inflammatory as well as oxidative stress. Neither IL-1 nor IL-6 increased in the EC supernatant following incubation with Mf. However, as *Wolbachia* spp. are an endosymbiotic bacteria of *Brugia malayi*, they produce heme [41]. Therefore, it is possible that *Wolbachia* spp. derived heme is a trigger of HO-1 production by HLMVEC. Additionally, HO-1 expression was repressed upon IFN-γ-stimulation of HLMVEC. Previous work has also shown that IFN-γ inhibits HO-1 in various cell types [42], [43] however, to the best of our knowledge the role of this mechanism has not been investigated in a functional context. While HO-1 has anti-inflammatory properties, Mf are also known to induce IFN-γ [44], [45], [46] thus the role HO-1 induction by Mf warrants further investigation.

Surprisingly, live intact Mf did not stimulate the expression of many angiogenic mediators including the key mediator, VEGF-A, in vascular EC, although, live Mf did stimulate pro-angiogenic COX-2 in HLMVEC. This is in line with previous work in which Simόn *et al.* found that *Wolbachia* surface protein from *D. immitis* and adult somatic antigen from *D. immitis*, both induce COX-2 in a human EC cell line [28], [29].

Mf also enhanced the surface expression of CCR5, which binds CCL3 (MIP-1α), CCL4 (MIP-1β) and CCL5 (RANTES), and mRNA expression of CCR10, which binds to CCL27 and CCL28 in vascular EC. The role of this increased expression of chemokine receptors is not clear, however, CCR5 itself is known to mediate angiogenesis [47].

Other work has investigated the potential of filarial antigen, and/or live worms, to initiate vessel dilatation and/or angiogenesis by measuring EC proliferation and tube formation *in vitro* [10], [28], [29], [48]. The results varied depending on differing use of HUVEC, lymphatic EC (LEC) or a human vascular EC cell line and parasite extracts or live nematode stages. For example, live female *B. malayi* decreased HUVEC proliferation [48] while LEC, but not HUVEC, cultured with adult *Brugia* or Mf extract showed increased proliferation [10] and *D. immitis* adult extract had no effect on the proliferation of a human EC cell line [29]. Live Mf and adults and their extracts all induced tube formation in LEC, however, in our experiments using live *B. malayi* Mf with vascular EC, both HUVEC and HLMVEC, we did not observe these structures [10].

In this study we report new insights into the EC response to live *B. malayi* Mf in their vascular environment, albeit within by the limitations of an *ex vivo* model which uses an EC isolate under static conditions incubated with parasites. Upon Mf exposure, extravasation of monocytes and neutrophils was partially blocked, while the transendothelial migration of lymphocytes was not altered. However, overall, Mf induced the expression of only a small number of cytokines, chemokines or pro-angiogenic mediators in human vascular EC. Furthermore, depletion of *Wolbachia* from live Mf did not significantly alter mRNA expression of these mediators. Taken together, our study suggests that live Mf are either relatively inert or that they are able to modulate local responses to promote their own survival and limit infection-induced pathology. Alternatively, Mf may induce a highly localised response mediated by other cells not present in this model system, rather than, a direct interaction between Mf and endothelium.

## Supporting Information

Figure S1 **CCL11 mRNA expression in HUVEC is not altered upon live Mf exposure.** HUVEC were cultured with live *B. malayi* Mf for 24 hours prior to isolation of total EC RNA. Real time qRT-PCR was used to analyse mRNA expression of CCL11 in HUVEC. Data are shown as the mean and standard deviation of three independent experiments and Students t-test was performed to analyse statistical significance.
(TIF)

Table S1 **Effect of Mf on cytokine, chemokine, chemokine receptor and further related mediator gene expression levels of HUVEC.** HUVEC were cultured (1×10^6^ cells/T25 flask) for 64.5 h before being co-cultured with *B. malayi* Mf (125,000/T25 flask). After 24 h of co-culture with or without Mf, total RNA from HUVEC was isolated and an oligo microarray was performed analysing the gene expression levels of cytokines, chemokines, chemokine receptors and related mediators. Data are shown as the mean and as the fold change in mRNA expression of all genes assessed compared to mRNA expression in unstimulated HUVEC. *Abbrevation nd = not determined*.
(DOC)

Table S2 **Effect of Mf on cytokine, chemokine and further related mediator gene expression levels of HLMVEC.** HLMVEC were cultured (1×10^6^ cells/T25 flask) for 64.5 h before being co-cultured with *B. malayi* Mf (125,000/T25 flask). After 24 h of co-culture with or without Mf, total RNA from HLMVEC was isolated and an oligo microarray was performed analysing the gene expression levels of cytokines, chemokines, chemokine receptors and related mediators. The data are shown as the result of one experiment.
(DOC)

Table S3 **Effect of Mf on cytokine protein expression levels of HUVEC.** HUVEC were cultured (1×10^6^ cells/T25 flask) for 64.5 h before being co-cultured with *B. malayi* Mf (125,000/T25 flask). After 24 h of co-culture with or without Mf, supernatant from HUVEC was harvested and secreted levels of cytokines were analysed with an antibody-based protein array. Data are shown as the mean per group of two independent experiments.
(DOC)

Table S4 **List of genes targeted for qRT-PCR used to evaluate gene expression.**
(DOC)

Table S5 **Analysis of gene expression using the oligo microarray for angiogenesis mediator genes.** HUVEC were cultured (1×10^6^ cells/T25 flask) for 64.5 h prior to co-culture with the absence or presence of either *Wolbachia*-depleted or *Wolbachia*-intact *B. malayi* Mf (125,000/T25 flask). After 24 h, total HUVEC RNA was isolated and analysed using an oligo microarray for mRNA expression of angiogenesis mediators. The data are presented as mean of expression units of two independent experiments normalised to β-actin and fold difference of the means. *Abbrevation nd = not determined*.
(DOC)

## References

[1] Hawking F (1968) The periodic migration of microfilariae of *Brugia malayi* and its response to various stimuli. American Journal of Tropical Medicine and Hygiene 17: 724–729. 4971019 10.4269/ajtmh.1968.17.724

[2] Hawking F, Thurston JP (1951) The periodicity of microfilariae. I. The distribution of microfilariae in the body. Transactions of the Royal Society of Tropical Medicine and Hygiene 45: 307–328. 14901442 10.1016/s0035-9203(51)80003-8

[3] Goodridge HS, Wilson EH, Harnett W, Campbell CC, Harnett MM, et al (2001) Modulation of macrophage cytokine production by ES-62, a secreted product of the filarial nematode *Acanthocheilonema viteae* . Journal of Immunology 167: 940–945. 10.4049/jimmunol.167.2.940 11441102

[4] Daehnel K, Gillette-Ferguson I, Hise AG, Diaconu E, Harling MJ, et al (2007) Filaria/*Wolbachia* activation of dendritic cells and development of Th1-associated responses is dependent on Toll-like receptor 2 in a mouse model of ocular onchocerciasis (river blindness). Parasite Immunology 29: 455–465. 17727569 10.1111/j.1365-3024.2007.00962.x

[5] Babu S, Blauvelt CP, Kumaraswami V, Nutman TB (2005) Diminished expression and function of TLR in lymphatic filariasis: a novel mechanism of immune dysregulation. J Immunol 175: 1170–1176. 16002719 10.4049/jimmunol.175.2.1170

[6] Magder S, Neculcea J, Neculcea V, Sladek R (2006) Lipopolysaccharide and TNF-alpha produce very similar changes in gene expression in human endothelial cells. Journal of Vascular Research 43: 447–461. 16921252 10.1159/000095162

[7] Nilsen EM, Johansen FE, Jahnsen FL, Lundin KE, Scholz T, et al (1998) Cytokine profiles of cultured microvascular endothelial cells from the human intestine. Gut 42: 635–642. 9659156 10.1136/gut.42.5.635 PMC1727090

[8] Debrah AY, Mand S, Specht S, Marfo-Debrekyei Y, Batsa L, et al (2006) Doxycycline reduces plasma VEGF-C/sVEGFR-3 and improves pathology in lymphatic filariasis. PLoS Pathogens 2: e92. 17044733 10.1371/journal.ppat.0020092 PMC1564427

[9] Debrah AY, Mand S, Toliat MR, Marfo-Debrekyei Y, Batsa L, et al (2007) Plasma vascular endothelial growth Factor-A (VEGF-A) and VEGF-A gene polymorphism are associated with hydrocele development in lymphatic filariasis. American Journal of Tropical Medicine and Hygiene 77: 601–608. 17978056

[10] Bennuru S, Nutman TB (2009) Lymphangiogenesis and lymphatic remodeling induced by filarial parasites: implications for pathogenesis. PLoS Pathogens 5: e1000688. 20011114 10.1371/journal.ppat.1000688 PMC2781552

[11] Roye O, Delhem N, Trottein F, Remoue F, Nutten S, et al (1998) Dermal endothelial cells and keratinocytes produce IL-7 in vivo after human *Schistosoma mansoni* percutaneous infection. Journal of Immunology 161: 4161–4168. 9780189

[12] Angeli V, Faveeuw C, Delerive P, Fontaine J, Barriera Y, et al (2001) *Schistosoma mansoni* induces the synthesis of IL-6 in pulmonary microvascular endothelial cells: role of IL-6 in the control of lung eosinophilia during infection. European Journal of Immunology 31: 2751–2761. 11536174 10.1002/1521-4141(200109)31:9<2751::aid-immu2751>3.0.co;2-4

[13] Jaffe EA, Nachman RL, Becker CG, Minick CR (1973) Culture of human endothelial cells derived from umbilical veins. Identification by morphologic and immunologic criteria. Journal of Clinical Investigation 52: 2745–2756. 4355998 10.1172/JCI107470 PMC302542

[14] Turner JD, Mand S, Debrah AY, Muehlfeld J, Pfarr K, et al (2006) A randomized, double-blind clinical trial of a 3-week course of doxycycline plus albendazole and ivermectin for the treatment of *Wuchereria bancrofti* infection. Clinical Infectious Diseases 42: 1081–1089. 16575724 10.1086/501351

[15] McGarry HF, Egerton GL, Taylor MJ (2004) Population dynamics of *Wolbachia* bacterial endosymbionts in *Brugia malayi* . Molecular and Biochemical Parasitology 135: 57–67. 15287587 10.1016/j.molbiopara.2004.01.006

[16] Al-Musawi SL, Lock F, Simbi BH, Bayol SAM, Stickland NC (2011) Muscle specific differences in the regulation of myogenic differentiation in chickens genetically selected for divergent growth rates. Differentiation 82: 127–135. 21723031 10.1016/j.diff.2011.05.012 PMC3181402

[17] Ou X, Thomas GR, Chacon MR, Tang L, Selkirk ME (1995) *Brugia malayi*: differential susceptibility to and metabolism of hydrogen peroxide in adults and microfilariae. Experimental Parasitology 80: 530–540. 7729488 10.1006/expr.1995.1065

[18] Thomas GR, McCrossan M, Selkirk ME (1997) Cytostatic and cytotoxic effects of activated macrophages and nitric oxide donors on *Brugia malayi* . Infection and Immunity 65: 2732–2739. 9199443 10.1128/iai.65.7.2732-2739.1997 PMC175385

[19] Taylor MJ, Cross HF, Mohammed AA, Trees AJ, Bianco AE (1996) Susceptibility of *Brugia malayi* and *Onchocerca lienalis* microfilariae to nitric oxide and hydrogen peroxide in cell-free culture and from IFN-g-activated macrophages. Parasitology 112: 315–322. 8728995 10.1017/s0031182000065835

[20] Simons JE, Rothenberg ME, Lawrence RA (2005) Eotaxin-1-regulated eosinophils have a critical role in innate immunity against experimental *Brugia malayi* infection. European Journal of Immunology 35: 189–197. 15593125 10.1002/eji.200425541

[21] Tang L, Ou X, Henkle-Dührsen J, Selkirk ME (1994) Extracellular and cytoplasmic Cu/Zn superoxide dismutases from lymphatic filarial nematode parasites. Infection and Immunity 62: 961–967. 8112870 10.1128/iai.62.3.961-967.1994 PMC186210

[22] Ou X, Tang L, McCrossan M, Henkle-Duhrsen K, Selkirk ME (1995) *Brugia malayi*: localisation and differential expression of extracellular and cytoplasmic CuZn superoxide dismutases in adults and microfilariae. Experimental Parasitology 80: 515–529. 7729487 10.1006/expr.1995.1064

[23] Ghosh I, Eisinger SW, Raghavan N, Scott AL (1998) Thioredoxin peroxidases from *Brugia malayi* . Molecular and Biochemical Parasitology 91: 207–220. 9566515 10.1016/s0166-6851(97)00213-2

[24] Ley K, Laudanna C, Cybulsky MI, Nourshargh S (2007) Getting to the site of inflammation: the leukocyte adhesion cascade updated. Nature Reviews Immunology 7: 678–689. 10.1038/nri2156 17717539

[25] Kilgore KS, Flory CM, Miller BF, Evans VM, Warren JS (1996) The membrane attack complex of complement induces interleukin-8 and monocyte chemoattractant protein-1 secretion from human umbilical vein endothelial cells. American Journal of Pathology 149: 953–961. 8780399 PMC1865152

[26] MacDonald AS, Loke P, Martynoga R, Dransfield I, Allen JE (2003) Cytokine-dependent inflammatory cell recruitment patterns in the peritoneal cavity of mice exposed to the parasitic nematode *Brugia malayi* . Medical Microbiology and Immunology 192: 33–40. 12592561 10.1007/s00430-002-0156-8

[27] Rees-Roberts D, Mullen LM, Gounaris K, Selkirk ME (2010) Inactivation of the complement anaphylatoxin C5a by secreted products of parasitic nematodes. International Journal for Parasitology 40: 527–532. 19874826 10.1016/j.ijpara.2009.10.006 PMC2852653

[28] Simon F, Morchon R, Rodriguez-Barbero A, Lopez-Belmonte J, Grandi G, et al (2008) *Dirofilaria immitis* and *Wolbachia*-derived antigens: its effect on endothelial mammal cells. Veterinary Parasitology 158: 223–231. 18922634 10.1016/j.vetpar.2008.09.010

[29] Morchon R, Rodriguez-Barbero A, Velasco S, Lopez-Belmonte J, Simon F (2008) Vascular endothelial cell activation by adult *Dirofilaria immitis* antigens. Parasitology International 57: 441–446. 18603468 10.1016/j.parint.2008.05.004

[30] Sartono E, Kruize YC, Kurniawan A, van der Meide PH, Partono F, et al (1995) Elevated cellular immune responses and interferon-gamma release after long-term diethylcarbamazine treatment of patients with human lymphatic filariasis. Journal of Infectious Diseases 171: 1683–1687. 7769319 10.1093/infdis/171.6.1683

[31] Turner JD, Langley RS, Johnston KL, Gentil K, Ford L, et al (2009) *Wolbachia* lipoprotein stimulates innate and adaptive immunity through Toll-like receptors 2 and 6 to induce disease manifestations of filariasis. Journal of Biological Chemistry 284: 22364–22378. 19458089 10.1074/jbc.M901528200 PMC2755959

[32] Turner JD, Langley RS, Johnston KL, Egerton G, Wanji S, et al (2006) *Wolbachia* endosymbiotic bacteria of *Brugia malayi* mediate macrophage tolerance to TLR- and CD40-specific stimuli in a MyD88/TLR2-dependent manner. Journal of Immunology 177: 1240–1249. 10.4049/jimmunol.177.2.1240 16818783

[33] Cross HF, Haarbrink M, Egerton G, Yazdanbakhsh M, Taylor MJ (2001) Severe reactions to filarial chemotherapy and release of *Wolbachia* endosymbionts into blood. Lancet 358: 1873–1875. 11741630 10.1016/S0140-6736(01)06899-4

[34] Keiser PB, Reynolds SM, Awadzi K, Ottesen EA, Taylor MJ, et al (2002) Bacterial endosymbionts of *Onchocerca volvulus* in the pathogenesis of posttreatment reactions. Journal of Infectious Diseases 185: 805–811. 11920298 10.1086/339344

[35] Camhi SL, Alam J, Otterbein L, Sylvester SL, Choi AM (1995) Induction of heme oxygenase-1 gene expression by lipopolysaccharide is mediated by AP-1 activation. American Journal of Respiratory Cell and Molecular Biology 13: 387–398. 7546768 10.1165/ajrcmb.13.4.7546768

[36] Gong P, Hu B, Stewart D, Ellerbe M, Figueroa YG, et al (2001) Cobalt induces heme oxygenase-1 expression by a hypoxia-inducible factor-independent mechanism in Chinese hamster ovary cells: regulation by Nrf2 and MafG transcription factors. Journal of Biological Chemistry 276: 27018–27025. 11356853 10.1074/jbc.M103658200

[37] Iyer S, Woo J, Cornejo MC, Gao L, McCoubrey W, et al (1998) Characterization and biological significance of immunosuppressive peptide D2702.75-84(E→V) binding protein. Isolation of heme oxygenase-1. Journal of Biological Chemistry 273: 2692–2697. 9446574 10.1074/jbc.273.5.2692

[38] Lee PJ, Jiang BH, Chin BY, Iyer NV, Alam J, et al (1997) Hypoxia-inducible factor-1 mediates transcriptional activation of the heme oxygenase-1 gene in response to hypoxia. Journal of Biological Chemistry 272: 5375–5381. 9038135

[39] Yang G, Nguyen X, Ou J, Rekulapelli P, Stevenson DK, et al (2001) Unique effects of zinc protoporphyrin on HO-1 induction and apoptosis. Blood 97: 1306–1313. 11222374 10.1182/blood.v97.5.1306

[40] Yeh LH, Alayash AI (2004) Effects of cell-free hemoglobin on hypoxia-inducible factor (HIF-1alpha) and heme oxygenase (HO-1) expressions in endothelial cells subjected to hypoxia. Antioxidants and Redox Signaling 6: 944–953. 15548892 10.1089/ars.2004.6.944

[41] Foster J, Ganatra M, Kamal I, Ware J, Makarova K, et al (2005) The *Wolbachia* genome of *Brugia malayi*: endosymbiont evolution within a human pathogenic nematode. PLoS Biology 3: e121. 15780005 10.1371/journal.pbio.0030121 PMC1069646

[42] Takahashi K, Nakayama M, Takeda K, Fujia H, Shibahara S (1999) Suppression of heme oxygenase-1 mRNA expression by interferon-gamma in human glioblastoma cells. Journal of Neurochemistry 72: 2356–2361. 10349844 10.1046/j.1471-4159.1999.0722356.x

[43] Udono-Fujimori R, Takahashi K, Takeda K, Furuyama K, Kaneko K, et al (2004) Expression of heme oxygenase-1 is repressed by interferon-gamma and induced by hypoxia in human retinal pigment epithelial cells. European Journal of Biochemistry 271: 3076–3084. 15233805 10.1111/j.1432-1033.2004.04241.x

[44] Lawrence RA, Allen JE, Gray CA (2000) Requirements for in vivo IFN-gamma induction by live microfilariae of the parasitic nematode, *Brugia malayi* . Parasitology 120: 631–640. 10874726 10.1017/s003118209900596x

[45] Gray CA, Lawrence RA (2002) Interferon-gamma and nitric oxide production are not required for the immune-mediated clearance of *Brugia malayi* microfilariae in mice. Parasite Immunology 24: 329–336. 12102718 10.1046/j.1365-3024.2002.00464.x

[46] Lawrence RA, Devaney E (2001) Lymphatic filariasis: parallels between the immunology of infection in humans and mice. Parasite Immunology 23: 353–361. 11472555 10.1046/j.1365-3024.2001.00396.x

[47] Wu Y, Li YY, Matsushima K, Baba T, Mukaida N (2008) CCL3-CCR5 axis regulates intratumoral accumulation of leukocytes and fibroblasts and promotes angiogenesis in murine lung metastasis process. Journal of Immunology 181: 6384–6393. 10.4049/jimmunol.181.9.6384 18941229

[48] Rao UR, Zometa CS, Vickery AC, Kwa BH, Nayar JK, et al (1996) Effect of *Brugia malayi* on the growth and proliferation of endothelial cells in vitro. Journal of Parasitology 82: 550–556. 8691362

