# Supplementary figures and images for: Live Brugia malayi Microfilariae Inhibit Transendothelial Migration of Neutrophils and Monocytes
Source: PLoS Negl Trop Dis. 2012 Nov 29;6(11):e1914. doi: 10.1371/journal.pntd.0001914 (PMC3510151; doi:10.1371/journal.pntd.0001914)

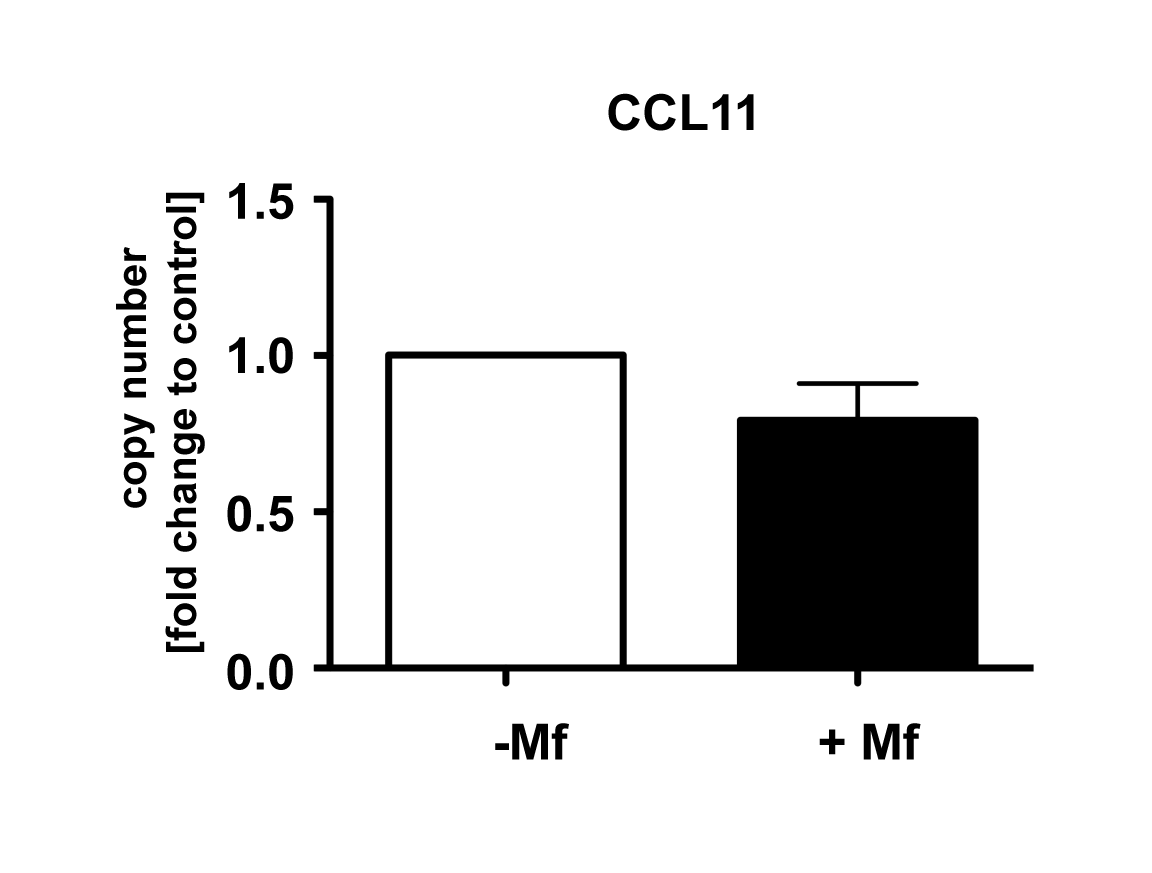

Supplement: Figure S1 — CCL11 mRNA expression in HUVEC is not altered upon live Mf exposure. HUVEC were cultured with live B. malayi Mf for 24 hours prior to isolation of total EC RNA. Real time qRT-PCR was used to analyse mRNA expression of CCL11 in HUVEC. Data are shown as the mean and standard deviation of three independent experiments and Students t-test was performed to analyse statistical significance. (TIF) [file pntd.0001914.s001.tif]
