# Supplementary material for: Live Brugia malayi Microfilariae Inhibit Transendothelial Migration of Neutrophils and Monocytes
Source: PLoS Negl Trop Dis. 2012 Nov 29;6(11):e1914. doi: 10.1371/journal.pntd.0001914 (PMC3510151; doi:10.1371/journal.pntd.0001914)
Supplement: Table S1 — Effect of Mf on cytokine, chemokine, chemokine receptor and further related mediator gene expression levels of HUVEC. HUVEC were cultured (1×10^6^ cells/T25 flask) for 64.5 h before being co-cultured with B. malayi Mf (125,000/T25 flask). After 24 h of co-culture with or without Mf, total RNA from HUVEC was isolated and an oligo microarray was performed analysing the gene expression levels of cytokines, chemokines, chemokine receptors and related mediators. Data are shown as the mean and as the fold change in mRNA expression of all genes assessed compared to mRNA expression in unstimulated HUVEC. Abbrevation nd = not determined. (DOC) [file pntd.0001914.s002.doc]

**Table 1:**

|  | HUVEC | HUVEC + Mf | Ratio  HUVEC + Mf/ HUVEC | Genbank |
| --- | --- | --- | --- | --- |
| C5 | 0.95 | 3.14 | 3.31 | NW 001735 |
| Chemokine binding protein 2 (CCBP2) | 1.2 | 1.04 | -0.87 | NM 001296 |
| CCL1 (I-309) | 1.34 | 3.065 | 2.29 | NM 002981 |
| CCL2 (MCP-1) | 0.19 | 0.25 | nd | NM 002982 |
| CCL3 (MIP-1α) | -0.09 | -0.01 | nd | NM 002983 |
| CCL4 (MIP-1β) | 0 | 0.1 | nd | NM 002984 |
| CCL4-like 1 (CCL4L1) | 0.38 | 0.66 | nd | NM 207007 |
| CCL5 (RANTES) | 1.04 | 1.66 | 1.6 | NM 002985 |
| CCL7 (MCP-3) | 0.95 | 2.93 | 3.08 | NM 006273 |
| CCL8 (MCP-2) | 0.92 | 1.83 | 1.99 | NM 005623 |
| CCL11 (eotaxin) | 0.5 | 0.4 | nd | NM 002986 |
| CCL13 (MCP-4) | 0.09 | 0.38 | nd | NM 005408 |
| CCL15 (MIP-1δ) | 0.33 | 0.8 | nd | NM 004167 |
| CCL16 (HCC-4) | 0.32 | 0.68 | nd | NM 004590 |
| CCL17 (TARC) | 0.37 | 0.77 | nd | NM 002987 |
| CCL18 (PARC) | 0.53 | 0.84 | nd | NM 002988 |
| CCL19 (MIP-3β) | 0.18 | 0.34 | nd | NM 006274 |
| CCL20 (MIP-3α) | 0.01 | 0.18 | nd | NM 004591 |
| CCL21 (6Ckine) | 0.04 | 0.31 | nd | NM 002989 |
| CCL23 (MIP-3) | 0.95 | 3.13 | 3.3 | NM 005064 |
| CCL24 (eotaxin-2) | 0.43 | 0.29 | nd | NM 002991 |
| CCL25 (TECK) | 0 | 0.13 | nd | NM 005624 |
| CCL26 (eotaxin-3) | 0 | 0.07 | nd | NM 006072 |
| CCL27 (CTACK) | 0.21 | 0.21 | nd | NM 006664 |
| CCL28 (MEC) | -0.06 | 0 | nd | NM 019846 |
| CCR1 | -0.06 | 0.01 | nd | NM 001295 |
| CCR2 | -0.06 | -0.03 | nd | NM 000648 |
| CCR3 | -0.06 | -0.01 | nd | NM 001837 |
| CCR4 | 0.15 | 0.56 | nd | NM 005508 |
| CCR5 | 0.94 | 3.17 | 3.37 | NM 000579 |
| CCR6 | 0.41 | 0.8 | nd | NM 004367 |
| CCR7 | 0.93 | 3.1 | 3.33 | NM 001838 |
| CCR8 | 0.93 | 3.06 | 3.29 | NM 005201 |
| CCR9 | 0.3 | 0.3 | nd | NM 006641 |
| CCR10 | 0.98 | 3.09 | 3.15 | NM 016602 |
| CCRL1 | -0.08 | -0.02 | nd | NM 016557 |
| CCRL2 | -0.04 | 0.01 | nd | NM003965 |
| Chemokine-like factor (CKLF) | 0.09 | 0.22 | nd | NM 181641 |
| CKLF-like MARVEL transmembrane domain containing 1 (CMTM1) | 0.07 | 0.36 | nd | NM 181269 |
| CMTM2 | 0.34 | 0.36 | nd | NM 144673 |
| CMTM3 | 0.34 | 0.1 | nd | NM 144601 |
| CMTM4 | 0.92 | 1.58 | 1.72 | NM 178818 |
| CMTM5 | 0.07 | 0.2 | nd | NM 138460 |
| CMTM6 | 0.58 | 1 | 1.72 | NM 017801 |
| CMTM7 | 0.3 | -0.06 | nd | NM 138410 |
| CMTM8 | -0.03 | 0.01 | nd | NM 178868 |
| Chemokine-like receptor 1 (CMKLR1) | -0.05 | -0.03 | nd | NM 004072 |
| Colony stimulating factor 3 (CSF3) | 1.21 | 1.04 | 0.86 | NM 000759 |
| CX3CL1 | 0.55 | 0.66 | nd | NM 002996 |
| CX3CR1 | 0 | 0.03 | nd | NM 001337 |
| CXCL1 (GROα) | 0.35 | 0.09 | nd | NM 001511 |
| CXCL2 (GROβ) | 0.71 | 0.78 | nd | NM 002089 |
| CXCL3 (GROγ) | 0.81 | 1.12 | 1.38 | NM 002090 |
| CXCL5 (ENA-78) | -0.03 | -0.02 | nd | NM 002994 |
| CXCL6 (GCP-2) | 0.06 | 0.05 | nd | NM 002993 |
| CXCL9 (MIG) | -0.06 | -0.01 | nd | NM 002416 |
| CXCL10 (IP-10) | 0.89 | 1.72 | 1.93 | NM 001565 |
| CXCL11 (I-TAC) | 1.09 | 1.79 | 1.64 | NM 005409 |
| CXCL12 (SDF-1) | 0.1 | -0.06 | nd | NM 000609 |
| CXCL13 (BCA-1) | -0.05 | -0.08 | nd | NM 006419 |
| CXCL14 (BRAK) | 0.94 | 2.22 | 2.36 | NM 004887 |
| CXCL16 | -0.01 | -0.02 | nd | NM 022059 |
| CXCR3 | 0.12 | 0.18 | nd | NM 001504 |
| CXCR4 | 0.26 | 0.21 | nd | NM 003467 |
| CXCR6 | 0.89 | 1.7 | nd | NM 006564 |
| CXCR7 | 0.09 | 0.11 | nd | NM 020311 |
| Cytoplasmic FMR1 interacting protein 2 (CYFIP2) | 0.97 | 3.04 | 3.13 | NM 014376 |
| Endothelial cell growth factor 1 (ECGF-1) | 0.84 | 0.83 | nd | NM 001953 |
| Erythropoietin (EPO) | 0.94 | 2.95 | 3.14 | NM 000799 |
| Duffy blood group chemokine receptor (DARC) | 0.53 | 0.65 | nd | NM 001296 |
| Growth differentiation factor 5 (GDF5) | 0.01 | 0.1 | nd | NM 000557 |
| G protein-coupled receptor 109B (GPR109B) | 0.98 | 3.09 | 3.15 | NM 006018 |
| G-protein-coupled receptor 31(GPR31) | 0.45 | 0.38 | nd | NM 005299 |
| G protein-coupled receptor 81 (GPR81) | 0.95 | 3.11 | 3.27 | NM 032554 |
| Hypoxia-inducible factor-1α (HIF-1α) | 1 | 2.5 | 2.5 | NM 001530 |
| IL-1α | 0.96 | 3.15 | 3.28 | NM 000575 |
| IL-4 | 0.53 | 0.7 | nd | NM 000589 |
| IL-8 | 0.01 | -0.08 | nd | NM 000584 |
| IL-8RA | 0.52 | 0.86 | nd | NM 000634 |
| IL-8RB | -0.11 | -0.08 | nd | NM 001557 |
| IL-13 | 1.15 | 2.76 | 2.4 | NM 002188 |
| IL-16 | -0.02 | 0.05 | nd | NM 004513 |
| IL-18 | 0.12 | 0.3 | nd | NM 001562 |
| Leukotriene B4 receptor (LTB4R) | -0.07 | -0.06 | nd | NM 181657 |
| MYD88 | 0.09 | 0.12 | nd | NM 002468 |
| NF-κB1 | 0.44 | 0.01 | nd | NM 003998 |
| Platelet factor 4 | 0.01 | -0.14 | nd | NM 002619 |
| Pro-platelet basic protein (PPBP) | 0.21 | 0.87 | nd | NM 002704 |
| Prolactin (PRL) | -0.11 | -0.12 | nd | NM 000948 |
| Regulator of G-protein signaling 3 (RGS3) | -0.11 | -0.14 | nd | NM 144488 |
| Regulator of G-protein signaling 13 (RGS13) | 0.07 | -0.11 | nd | NM 002927 |
| Small inducible cytokine subfamily E, member 1  (SCYE1) | 0.18 | -0.06 | nd | NM 004757 |
| Stromal cell-derived factor 2 (SDF2) | 0.42 | 1.03 | 2.45 | NM 006923 |
| Slit homologue 2 (SLIT2) | 0.11 | 0.26 | nd | NM 004787 |
| T-complex 10 (TCP-10) | -0.068 | -0.12 | nd | NM 004610 |
| TLR2 | -0.11 | -0.12 | nd | NM 003264 |
| TLR4 | -0.04 | -0.01 | nd | NM 003266 |
| TNF | 0.12 | 0.48 | nd | NM 000594 |
| Tumour necrosis factor receptor superfamily, member 12α (TNFRSF1α) | -0.01 | 0.29 | nd | NM 001065 |
| Tumour necrosis factor superfamily member 14 (TNFSF14) | -0.08 | -0.09 | nd | NM 003807 |
| Triggering receptor expressed on myeloid cells 1 (TREM1) | 0.72 | 1.92 | 2.67 | NM 018643 |
| TREM2 | 0.27 | -0.05 | nd | NM 018965 |
| XCL1 | 0.43 | 0.29 | nd | NM 002995 |
| XCR1 | 0.63 | 1.34 | 2.13 | NM 005283 |
