# Supplementary material for: Live Brugia malayi Microfilariae Inhibit Transendothelial Migration of Neutrophils and Monocytes
Source: PLoS Negl Trop Dis. 2012 Nov 29;6(11):e1914. doi: 10.1371/journal.pntd.0001914 (PMC3510151; doi:10.1371/journal.pntd.0001914)
Supplement: Table S3 — Effect of Mf on cytokine protein expression levels of HUVEC. HUVEC were cultured (1×10^6^ cells/T25 flask) for 64.5 h before being co-cultured with B. malayi Mf (125,000/T25 flask). After 24 h of co-culture with or without Mf, supernatant from HUVEC was harvested and secreted levels of cytokines were analysed with an antibody-based protein array. Data are shown as the mean per group of two independent experiments. (DOC) [file pntd.0001914.s004.doc]

**Table 3:**

|  | HUVEC | HUVEC + Mf | Ratio HUVEC + Mf/ HUVEC | GeneBank |
| --- | --- | --- | --- | --- |
| G-CSF | 0 | 0 | 0 | NM 172220 |
| GM-CSF | 0 | 0 | 0 | NM 000758 |
| GRO | 7.17 | 12.42 | 1.73 | NM 001511  NP 002080.1  NP 002081.2 |
| GRO-α | 6.68 | 9.38 | 1.4 | NM 001511 |
| IL-1α | 0 | 0 | 0 | NM 000575 |
| IL-2 | 0 | 0 | 0 | NM 000586 |
| IL-3 | 0.06 | 0 | 0 | NM 000588 |
| IL-5 | 0 | 0 | 0 | NM 000879 |
| IL-6 | 10.2 | 13.86 | 1.36 | NM 000600 |
| IL-7 | 3.09 | 3.08 | 1 | NM 000880 |
| IL-8 | 7.6 | 10.29 | 1.35 | NM 000584 |
| IL-10 | 1.48 | 2.01 | 1.36 | NM 000572 |
| IL-13 | 0.22 | 0.35 | 1.59 | NM 002188 |
| IL-15 | 0.14 | 0.36 | 2.57 | NM 000585 |
| IFN-γ | 0.03 | 0 | 0 | NM 000619 |
| MCP-1 | 10.79 | 11.92 | 1.1 | NM 002982 |
| MCP-2 | 0.29 | 1.33 | 4.59 | NM 005623 |
| MCP-3 | 0.9 | 0.91 | 1.01 | NM 006273 |
| MIG | 0.48 | 0.04 | 0.08 | NM 002416.1 |
| RANTES | 0.44 | 0.28 | 0.64 | NM 002985 |
| TGF-β1 | 0.12 | 0.33 | 2.75 | NM 000660 |
| TNF-α | 0.02 | 0.41 | 20.5 | NP 000585.2 |
| TNF-β | 0.04 | 0 | 0 | NP 000586.2 |
