# Supplementary material for: Live Brugia malayi Microfilariae Inhibit Transendothelial Migration of Neutrophils and Monocytes
Source: PLoS Negl Trop Dis. 2012 Nov 29;6(11):e1914. doi: 10.1371/journal.pntd.0001914 (PMC3510151; doi:10.1371/journal.pntd.0001914)
Supplement: Table S4 — List of genes targeted for qRT-PCR used to evaluate gene expression. (DOC) [file pntd.0001914.s005.doc]

Table 4

| Gene | Forward | Reverse | Product  length | Accession  number |
| --- | --- | --- | --- | --- |
| CCL1 | tctccagatgttgcttctca | tctttgcctctcttcagctt | 124 | NM_002981.1 |
| CCL11 | cccagaaagctgtgatcttc | tggagatttttggtccagat | 107 | NM_002986.2 |
| CCL23 | ccccagtgataagcaagttc | gaaggtagttgaggcaaga | 138 | NM_145898.1 |
| CCR10 | gagacccacagtctctcctg | cctgcctctttctcagtgtt | 110 | NM_016602.2 |
| IL -1α | tctccattcccaaacttagc | atatgcccaaggtgtgtctt | 103 | NM_000575.3 |
| Ang2 | ttgagaacagcaaactgcat | tgcaggtgctatggtcttta | 132 | NM_001147.2 |
| TNFSF15 | aaaaggctgaagagagcaaa | atcctgacccgagtagatca | 111 | NM_005118.2 |
| C5 | agtcactgaggctgacgttt | tgtgacttgagcaattccattt | 124 | NM_001735.2 |
| BAI1 | agctgcagcagtttgggttc | gtaggaggaggacacgcaga | 137 | NM-001702.2 |
| COX2 | ccacttcaagggattttgga | gagaaggcttcccagctttt | 150 | NM_000963.2 |
