# Supplementary material for: Live Brugia malayi Microfilariae Inhibit Transendothelial Migration of Neutrophils and Monocytes
Source: PLoS Negl Trop Dis. 2012 Nov 29;6(11):e1914. doi: 10.1371/journal.pntd.0001914 (PMC3510151; doi:10.1371/journal.pntd.0001914)
Supplement: Table S5 — Analysis of gene expression using the oligo microarray for angiogenesis mediator genes. HUVEC were cultured (1×10^6^ cells/T25 flask) for 64.5 h prior to co-culture with the absence or presence of either Wolbachia-depleted or Wolbachia-intact B. malayi Mf (125,000/T25 flask). After 24 h, total HUVEC RNA was isolated and analysed using an oligo microarray for mRNA expression of angiogenesis mediators. The data are presented as mean of expression units of two independent experiments normalised to β-actin and fold difference of the means. Abbrevation nd = not determined. (DOC) [file pntd.0001914.s006.doc]

**Table 5:**

|  | HUVEC | HUVEC + intact  Mf | HUVEC + *Wolbachia*-depleted Mf | Ratio: HUVEC + intact  Mf/ HUVEC | Ratio:  HUVEC +  *Wolbachia*-depleted Mf/ HUVEC | Ratio:  HUVEC +  intact  Mf/ HUVEC + *Wolbachia*-depleted Mf | GeneBank |
| --- | --- | --- | --- | --- | --- | --- | --- |
| Angiogenic factor  with G patch and  FHA domain 1 | 1.79 | 1.28 | 3.65 | nd | nd | 0.35 | NM 018046 |
| Angiogenin | 1.28 | 1.02 | 0.09 | nd | nd | nd | NM 001145 |
| Angiopoietin 1 | 1.97 | 2.13 | 0.548 | nd | nd | nd | NM 001146 |
| Angiopoietin 2 | 5.11 | 6.029 | 1.84 | 1.18 | 0.36 | 3.28 | NM 001147 |
| Angiopoietin-like 3 | 0.88 | 0.71 | 0.34 | nd | nd | nd | NM 014495 |
| Angiopoietin-like 4 | 1.78 | 1.14 | 1.12 | nd | nd | nd | NM 016109 |
| Alanyl (membrane)  aminopeptidase | 8.3 | 7.36 | 9 | 0.89 | 1.08 | 0.82 | NM 001150 |
| Brain-specific angiogenesis inhibitor 1 | 5.83 | 1.87 | 1.25 | 0.32 | 0.21 | 1.5 | NM 001702 |
| CCL2 | 5.1 | 7.6 | 6.86 | 1.49 | 1.35 | 1.11 | NM 002982 |
| CCL11 | 3.04 | 3.34 | 1.31 | 1.1 | 0.43 | 2.55 | NM 002986 |
| Coagulation factor II  (thrombin) | 0.12 | 0.23 | 0.17 | nd | nd | nd | NM 000506 |
| Collagen type XVIII  alpha 1 | 8.14 | 7.64 | 8.94 | 0.94 | 1.1 | 0.85 | NM 030582 |
| Collagen type IV  alpha 3 | 1.6 | 2.62 | 0.97 | nd | nd | nd | NM 000091 |
| Colony stimulating  factor 3 (CSF3) | 2.17 | 3.09 | 1.5 | 1.42 | nd | 1.06 | NM 000759 |
| CXCL1 | 2.35 | 3.66 | 3.17 | 1.56 | 1.35 | 1.15 | NM 001511 |
| CXCL2 | 2.44 | 2.8 | 1.05 | nd | nd | nd | NM 002089 |
| CXCL3 | 1.08 | 1.19 | 0.63 | nd | nd | nd | NM 002090 |
| CXCL5 | 2.99 | 2.29 | 1.06 | nd | nd | nd | NM 002994 |
| CXCL6 | 0.45 | 0.33 | 0.08 | nd | nd | nd | NM 002993 |
| CXCL9 | 0.56 | 0.49 | 0.33 | nd | nd | nd | NM 002416 |
| CXCL10 | 1.07 | 0.48 | 0.68 | nd | nd | nd | NM 001565 |
| CXCL11 | 1.33 | 1.67 | 0.71 | nd | nd | nd | NM 005409 |
| Endoglin | 8.3 | 7.47 | 8.76 | 0.9 | 1.05 | 0.85 | NM 000118 |
| Endothelial cell  growth factor 1  (platelet-derived) | 4.04 | 3.89 | 3.72 | 0.96 | 0.92 | 1.05 | NM 001953 |
| Endothelial  differentiation  sphingolipid G-  protein-coupled receptor 1 | 2.06 | 1.2 | 0.81 | nd | nd | nd | NM 001400 |
| Ephrin-A1 | 5.64 | 7.6 | 7.23 | 1.35 | 1.28 | 1.05 | NM 182685 |
| Ephrin-A2 | 4.32 | 5.35 | 4.05 | 1.24 | 0.94 | 1.32 | NM 001405 |
| Ephrin-A3 | 4.5 | 5.69 | 3.76 | 1.26 | 0.84 | 1.51 | NM 004952 |
| Ephrin-A5 | 0.75 | 0.23 | 0.09 | nd | nd | nd | NM 001962 |
| Ephrin-B2 | 2.8 | 2.47 | 1.19 | nd | nd | nd | NM 004093 |
| Epidermal growth  factor | 1.73 | 1.78 | 1.78 | nd | nd | nd | NM 001963 |
| Endothelial PAS  domain protein 1 | 3.96 | 5.75 | 3.44 | 1.45 | 0.87 | 1.67 | NM 001430 |
| Ephrin receptor B4 | 0.4 | 1.61 | 0.4 | nd | nd | nd | NM 004444 |
| Epiregulin | 0.26 | 0.76 | 0.26 | nd | nd | nd | NM 001432 |
| FGF-1 | 0.04 | 0.08 | 0.16 | nd | nd | nd | NM 000800 |
| FGF-2 | 0.22 | 0.06 | 0.5 | nd | nd | nd | NM 002006 |
| Fibroblast Growth  Factor 6 | 0.4 | 0.31 | 0.73 | nd | nd | nd | NM 020996 |
| Fibroblast Growth  Factor receptor 3 | 1.12 | 2.04 | 1.5 | nd | nd | nd | NM 000142 |
| Heart and neural  crest derivatives  expressed 2 | 0.25 | 0.61 | 0.25 | nd | nd | nd | NM 021973 |
| Heparanase | -0.05 | -0.01 | 0.01 | nd | nd | nd | NM 006665 |
| Hepatocyte growth  factor | 0.18 | 0.31 | 0.46 | nd | nd | nd | NM 000601 |
| Inhibitor of DNA  binding 1 | 0.43 | 0.59 | -0.02 | nd | nd | nd | NM 002165 |
| Inhibitor of DNA  binding 3 | 0.61 | 0.64 | 0.16 | nd | nd | nd | NM 002167 |
| IFN-α1 | 0.91 | 1.15 | 0.57 | nd | nd | nd | NM 024013 |
| IFN-β1 | 0.45 | 1.15 | 0.27 | nd | nd | nd | NM 002176 |
| IFN-γ | 0.34 | 1.24 | 0.27 | nd | nd | nd | NM 000619 |
| Insulin-like growth  factor 1 | 0.18 | 0.36 | 0.21 | nd | nd | nd | NM 000618 |
| IL-1β | 1.79 | 2.3 | 1.53 | nd | nd | nd | NM 000576 |
| IL-6 | 1.19 | 1.08 | 0.23 | nd | nd | nd | NM 000600 |
| IL-8 | 1.33 | 2.83 | 0.25 | nd | nd | nd | NM 000584 |
| IL-10 | 0.34 | 0.2 | 0.03 | nd | nd | nd | NM 000572 |
| IL-12A | 1.32 | 0.64 | 0.2 | nd | nd | nd | NM 000882 |
| IL-18 | 0.46 | 0.15 | 0 | nd | nd | nd | NM 001562 |
| Integrin α_v_ | 0.49 | 1.08 | 0.3 | nd | nd | nd | NM 002210 |
| Integrin β3 | 2.33 | 2.66 | 3.1 | nd | 1.33 | 0.86 | NM 000212 |
| Jagged 1 | 2.02 | 1.28 | 0.3 | nd | nd | nd | NM 000214 |
| Laminin α5 | 4.29 | 7.27 | 4.84 | 1.69 | 1.13 | 1.5 | NM 005560 |
| Leukocyte cell  derived  chemotaxin 1 | 0.37 | 0.48 | 0.22 | nd | nd | nd | NM 007015 |
| Leptin | 0.49 | 0.36 | 0.71 | nd | nd | nd | NM 000230 |
| Midkine | 0.7 | 1.8 | 0.27 | nd | nd | nd | NM 002391 |
| Matrix  metallopeptidase 2 | 8.13 | 7.5 | 8.77 | 0.92 | 1.08 | 0.86 | NM 004530 |
| Matrix metallopeptidase 9 | 0.77 | 1.07 | 0.53 | nd | nd | nd | NM 004994 |
| Matrix metallopeptidase 19 | 0.61 | 1.49 | 0.7 | nd | nd | nd | NM 002429 |
| Notch homolog 4  (*Drosophila*) | 2.53 | 3.6 | 1.15 | nd | nd | nd | NM 004557 |
| Natriuretic peptide  precursor B | 0.15 | 0.44 | -0.05 | nd | nd | nd | NM 002521 |
| Natriuretic peptide  receptor A/  guanyllate cyclase A | 0.69 | 0.34 | 0.054 | nd | nd | nd | NM 000906 |
| Neuropilin 1 | 5.75 | 7.27 | 7.34 | 1.26 | 1.28 | 0.99 | NM 003873 |
| Neuropilin 2 | 0.83 | 2 | 0.43 | nd | nd | nd | NM 003872 |
| Nudix | 0.71 | 1.41 | 0.38 | nd | nd | nd | NM 007083 |
| Platelet-derived growth factor-α | 1.2 | 1.81 | 1 | nd | nd | nd | NM 002607 |
| Platelet-derived growth factor-β | 5.65 | 7.43 | 7.71 | 1.32 | 1.36 | 0.96 | NM 002608 |
| Platelet factor 4 | 0.67 | 0.36 | -0.06 | nd | nd | nd | NM 002619 |
| Placental Growth  Factor | 4.38 | 6.98 | 4.62 | 1.59 | 1.05 | 1.51 | NM 002632 |
| Plasminogen activator | 5.31 | 7.3 | 6.66 | 1.37 | 1.25 | 1.1 | NM 002658 |
| Plasminogen | 0.46 | 0.97 | 0.35 | nd | nd | nd | NM 000301 |
| Plexin domain  containing 1 | 0.49 | 0.77 | 0.19 | nd | nd | nd | NM 020405 |
| Prokineticin 2 | 0.36 | 0.52 | 0.04 | nd | nd | nd | NM 021935 |
| Phosphatase and  tensin homolog  (PTEN) | 0.49 | 0.87 | 0.13 | nd | nd | nd | NM 000314 |
| Prostaglandin-endoperoxide synthase 1 | 2.37 | 1.69 | 0.98 | nd | nd | nd | NM 000962 |
| Prostaglandin-endoperoxide synthase 2 | 3.86 | 0.035 | 0.43 | 0.01 | 0.11 | nd | NM 000963 |
| Pleiotrophin | 1.28 | 0.05 | -0.01 | nd | nd | nd | NM 002825 |
| Serine proteinase  inhibitor | 0.7 | 0.75 | 0.53 | nd | nd | nd | NM 002615 |
| SH2 domain protein  2A | 0.64 | 0.88 | 0.25 | nd | nd | nd | NM 003975 |
| Sphingosine kinase  1 | 0.81 | 0.75 | 0.42 | nd | nd | nd | NM 021972 |
| Stabilin 1 | 0.35 | 0.46 | 0.34 | nd | nd | nd | NM 015136 |
| Stabilin 2 | 0.27 | 0.23 | 0.17 | nd | nd | nd | NM 017564 |
| TEK tyrosine kinase | 2.15 | 1.1 | 0.29 | nd | nd | nd | NM 000459 |
| TGF-α | 2.47 | 1.34 | 0.37 | nd | nd | nd | NM 003236 |
| TGF-β1 | 1.2 | 0.8 | 0.12 | nd | nd | nd | NM 000660 |
| TGF-β2 | 0.69 | 1 | 0.63 | nd | nd | nd | NM 003238 |
| TGF-β3 | 0.74 | 0.84 | 0.41 | nd | nd | nd | NM 003239 |
| Transforming growth factor  receptor-β1 | 0.67 | 0.6 | 0.4 | nd | nd | nd | NM 004612 |
| Thrombospondin 1 | 4.19 | 6.33 | 6.45 | 1.51 | 1.54 | 0.98 | NM 003246 |
| Thrombospondin 2 | 0.76 | 1.17 | 0.6 | nd | nd | nd | NM 003247 |
| Tie1 | 8.28 | 7.43 | 8.97 | 0.9 | 1.08 | 0.83 | NM 005424 |
| TIMP metallopeptidase inhibitor 1 | 8.32 | 7.43 | 8.94 | 0.89 | 1.07 | 0.83 | NM 003254 |
| TIMP metallopeptidase inhibitor 2 | 2.01 | 2.94 | 0.99 | nd | nd | nd | NM 003255 |
| TIMP metalopeptidase inhibitor 3 | 1.58 | 1.98 | 1.8 | nd | nd | nd | NM 000362 |
| TNF | 0.57 | 1.38 | 1.38 | nd | nd | nd | NM 000594 |
| TNF-α-induced protein 2 | 0.52 | 0.98 | 0.44 | nd | nd | nd | NM 006291 |
| Tumor Necrosis  Factor Receptor  Superfamily  Member 12A  (TNFRSF12A) | 3.34 | 5.79 | 2.18 | 1.73 | 0.65 | 2.66 | NM 016639 |
| Tumor Necrosis  Factor Superfamily  Member 15  (TNFRSF15) | 0.91 | 4.07 | 0.5 | 4.47 | nd | 8.14 | NM 005118 |
| Troponin T type 1 | 0.73 | 1.51 | 0.81 | nd | nd | nd | NM 003283 |
| VE-cadherin | 5.38 | 7.6 | 5.68 | 1.41 | 1.06 | 1.34 | NM 001795 |
| VEGF-A | 1.28 | 1.84 | 1.01 | nd | nd | nd | NM 003376 |
| VEGF-B | 8.06 | 7.37 | 8.94 | 0.91 | 1.11 | 0.82 | NM 003377 |
| VEGF-C | 3.31 | 3.83 | 4.56 | 1.16 | 1.38 | 0.84 | NM 005429 |
| VEGF-D | 1.01 | 2.95 | 1.07 | nd | nd | nd | NM 004469 |
| VEGFR1 | 0.3 | 0.88 | 0.27 | nd | nd | nd | NM 002019 |
| VEGFR2 | 1.88 | 1.33 | 0.19 | nd | nd | nd | NM 002253 |
